# Primase is required for helicase activity and helicase alters the specificity of primase in the enteropathogen *Clostridium difficile*

**DOI:** 10.1098/rsob.160272

**Published:** 2016-12-21

**Authors:** Erika van Eijk, Vasileios Paschalis, Matthew Green, Annemieke H. Friggen, Marilynn A. Larson, Keith Spriggs, Geoffrey S. Briggs, Panos Soultanas, Wiep Klaas Smits

**Affiliations:** 1 Department of Medical Microbiology, Leiden University Medical Center, Leiden, The Netherlands; 2 School of Chemistry, Center for Biomolecular Sciences, University of Nottingham, UK; 3 School of Pharmacy, University of Nottingham, UK; 4 Department of Pathology and Microbiology, University of Nebraska Medical Center, Omaha, NE 68198-5900, USA; 5 National Strategic Research Institute, Omaha, NE 68105, USA

**Keywords:** DNA replication initiation, helicase loading and activation, primase trinucleotide specificity, ATPase, *Clostridium difficile*

## Abstract

DNA replication is an essential and conserved process in all domains of life and may serve as a target for the development of new antimicrobials. However, such developments are hindered by subtle mechanistic differences and limited understanding of DNA replication in pathogenic microorganisms. *Clostridium difficile* is the main cause of healthcare-associated diarrhoea and its DNA replication machinery is virtually uncharacterized. We identify and characterize the mechanistic details of the putative replicative helicase (CD3657), helicase-loader ATPase (CD3654) and primase (CD1454) of *C. difficile*, and reconstitute helicase and primase activities *in vitro*. We demonstrate a direct and ATP-dependent interaction between the helicase loader and the helicase. Furthermore, we find that helicase activity is dependent on the presence of primase *in vitro*. The inherent trinucleotide specificity of primase is determined by a single lysine residue and is similar to the primase of the extreme thermophile *Aquifex aeolicus.* However, the presence of helicase allows more efficient de novo synthesis of RNA primers from non-preferred trinucleotides. Thus, loader–helicase–primase interactions, which crucially mediate helicase loading and activation during DNA replication in all organisms, differ critically in *C. difficile* from that of the well-studied Gram-positive *Bacillus subtilis* model.

## 1. Background

Extensive research, primarily on the model organisms *Escherichia coli* (Gram- negative) and *Bacillus subtilis* (Gram-positive), has shown that many different proteins are involved in DNA replication. Although the overall mechanism of replication is highly conserved in all domains of life and replication is initiated by the highly conserved DNA replication initiator protein DnaA in all bacteria [1], it is perhaps not surprising that details of the molecular mechanisms can vary substantially as these prokaryotes diverged more than 3 billion years ago [2].

One of the best-characterized distinctions between *B. subtilis* and *E. coli* is the mechanism of loading the replicative helicase at the origin of replication (*oriC*), an essential step in the DNA replication process of bacteria [3,4]. Helicase is required to unwind the DNA duplex at the replication fork, and during the loading step a functional helicase multimer is assembled onto the DNA. In the Enterobacteria, Firmicutes and Aquificae helicase loading is facilitated by a specific loader protein, which is not conserved in bacteria outside these phyla [5]. However, the strategy of helicase loading among bacteria that do encode a loader protein also differs [6,7].

For historical reasons, the nomenclature for the replication proteins differs between bacterial species (e.g. *E. coli* helicase, DnaB; *B. subtilis* helicase, DnaC). For clarity, protein names hereafter will be used in conjunction with species and either written in full or abbreviated (e.g. Ec and Bs). The *E. coli* helicase (EcDnaB) is loaded by a single loader protein (EcDnaC) *in vivo* [8–10], whereas loading of the *B. subtilis* helicase (BsDnaC) requires three accessory proteins (BsDnaD, BsDnaB and BsDnaI) *in vivo* [11–14] in addition to the replication initiator DnaA that is required in both organisms. One possible explanation for the requirement of multiple proteins in *B. subtilis* may lie in the fact that *E. coli* and *B. subtilis* employ different mechanisms to deliver the replicative helicase onto the DNA [6,7]. Alternatively, it may reflect different *oriC* architectures, requiring different mechanisms of origin remodelling [15].

Replicative helicases form hexameric rings and require single-stranded DNA (ssDNA) to be threaded through the central channel of the protein to unwind the DNA duplex [7,16]. To accomplish this, it is thought that either the pre-formed ring is physically opened (ring-breaker) or that the ring is assembled from monomers at *oriC* (ring-maker) [6].

In *E. coli*, pre-formed hexamers of the helicase protein are capable of self-loading onto ssDNA. They display *in vitro* translocation and unwinding activities, which are highly induced in the presence of the loader protein [8]. This is in contrast with *B. subtilis*, where pre-assembled hexameric helicase is inactive, irrespective of the presence of the loader protein. *In vitro*, *B. subtilis* helicase activity is only observed when the helicase protein is monomeric and the loader protein is present [17]. Thus, helicase loading in *E. coli* is an example of the ring-breaker mechanism, whereas the situation in *B. subtilis* exemplifies a ring-maker mechanism [6].

*Bacillus subtilis* helicase loading *in vivo* is a hierarchical process [12–14]. Initially, the double-stranded DNA (dsDNA) at *oriC* is melted into ssDNA by the initiation protein DnaA, thereby creating a substrate for primosome assembly. The BsDnaD and BsDnaB co-loader proteins, which are structural homologues (PFAM DnaB_2), associate sequentially with the replication origin [12] and possibly contribute to origin remodelling. This ultimately enables the ATPase loader protein to load the helicase [12–15,18–22].

The replication initiation protein (BsDnaA) and helicase-loader protein (BsDnaI) belong to the AAA+ (ATPases associated with various cellular activities) family of ATPases [23–26]. These AAA+ enzymes are, in their turn, part of the additional strand catalytic glutamate (ASCE) family [15,26–30]. The BsDnaI loader protein consists of a C-terminal AAA+ domain that is necessary for nucleotide and ssDNA binding, and an N-terminal helicase-interacting domain [17]. The loader interacts with the helicase regulating its activity and is therefore pivotal in replisome assembly [4,31,32].

The BsDnaC helicase is also an ASCE protein, and belongs to RecA-type helicase Superfamily 4 (SF4), which is involved in DNA replication [28,33,34]. The SF4 superfamily of helicases is characterized by five sequence motifs: H1, H1a, H2, H3 and H4 [28,35,36]. Motifs H1 and H2 are equivalent to the ATP-coordinating Walker A and B motifs found in many other ATPases [28].

The multi-protein primosome of *B. subtilis* consists not only of the helicase-loader protein and helicase, but also of primase [37,38]. Primase is pivotal for the initiation of DNA synthesis at the replication origin and remains of utmost importance during the DNA-replication process in restarting stalled replication forks as well as de novo priming of Okazaki fragments for lagging strand synthesis [39]. Prokaryotic primases have a three-domain structure consisting of an N-terminal zinc-binding domain (ZBD), a central RNA polymerase domain that catalyses the polymerization of ribonucleotides and a C-terminal domain that either is responsible for the interaction with helicase (helicase interaction domain) or has helicase activity itself [40]. The latter region, also known as P16 (reflecting the approximate mass of 16 kDa), is variable in prokaryotes and seems to be crucial for the direct interaction with and activation of the helicase [41–44]. Interestingly, the P16 domain is structurally and functionally homologous to the N-terminal domain of the replicative helicase to which it binds [43,45,46]. Depending on the bacterial species, the interaction between helicase and primase can be either transient (*E. coli*) or stable (*Geobacillus stearothermophilus*) [41,47].

Primase and helicase affect each other's activities [41,44,48–50]. Helicase affects primase by modulating initiation specificity, stimulating primer synthesis, reducing the length of primers synthesized and increasing its affinity for single-stranded DNA [40,44,48–55]. Although increased activity of primase in the presence of helicase was shown for Firmicutes such as *Staphylococcus aureus* and *B. subtilis*, primer length was minimally or not altered in either organism [50,56,57]. Conversely, ATPase and helicase activities of helicase are stimulated by primase due to stabilization of the helicase hexamer by this interaction [41,44,58–60]. In *Helicobacter pylori*, it was shown that the interaction of primase with helicase resulted in dissociation of the double hexamer of helicase, thereby increasing ATP hydrolysis, DNA binding and unwinding [16].

Although no interaction of primase with loader protein has been demonstrated to date, it has been suggested that primase affects the interaction between helicase and the loader protein in *E. coli* [61]. Dissociation of loader protein from the C-terminal region of helicase in this organism is thought to be induced by primer synthesis and conformational changes resulting from primase–helicase interaction [61]. Similar observations were made in *B. subtilis*, where the loader protein was found to dissociate from a complex when primase and polymerase bind to helicase in gel-filtration experiments [57]. However, a ternary complex comprising helicase, loader and the helicase binding domain of primase in *G. stearothermophilus* is capable of loading [62]. These observations indicate that primosome formation, like helicase loading, may also be species-specific [59,61,62].

We are interested in DNA replication in the Gram-positive enteropathogen *C. difficile,* the most common causative agent of antibiotic associated diarrhoea [63,64]. Like *B. subtilis*, *C. difficile* belongs to the low-GC content Firmicutes. This strictly anaerobic bacterium can cause potentially fatal intestinal inflammation (colitis), is resistant to multiple antibiotics and is capable of forming highly resistant endospores [63,64]. However, the *C. difficile* DNA replication machinery is virtually uncharacterized.

Herein, we address this gap by identifying and characterizing the *C. difficile* replicative helicase (CD3657), helicase loader (CD3654) and primase (CD1454), and reconstituting helicase and primase activities *in vitro*. Our results indicate that the interaction between *C. difficile* helicase and primase is crucial for the functions of both proteins and that a single residue can strikingly determine trinucleotide specificity. Our results show that helicase loading and activation, as well as primase template specificity, in *C. difficile* differs critically from the *B. subtilis* model.

## 2. Results

### 2.1. *In silico* identification of putative replication initiation proteins

In *B. subtilis*, replication initiation requires the coordinated action of multiple proteins, DnaA, DnaD, DnaB and DnaI, *in vivo* [12,15]. BLASTP queries of the genome of *C. difficile* 630 (Genbank AM180355.1) using the amino acid sequence of these proteins from *B. subtilis* subsp. *subtilis* strain 168 (GenBank NC0989.1) allowed the identification of homologues of most, but not all, proteins that were found to be essential for replication initiation in *B. subtilis*.

**Figure 1.**
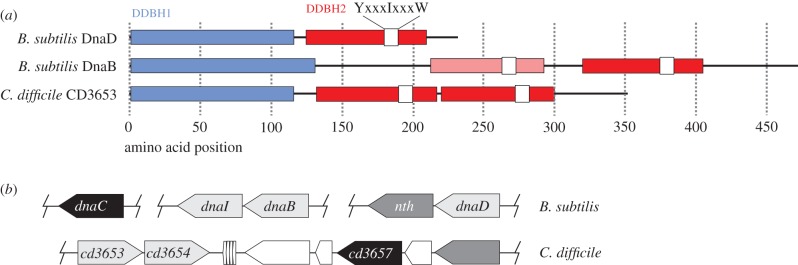
*In silico* analysis of putative replication initiation proteins of *C. difficile*. (*a*) Domain structure of BsDnaB, BsDnaD and CD3653. Domain nomenclature according to Marston *et al*. [18]. Note that DDBH2 corresponds to PFAM DnaB_2. (*b*) Chromosomal organization of the *dnaBI* genomic region of *B. subtilis* and the CD3653-CD3654 genomic region of *C. difficile*.

Homologues of the initiation protein DnaA (CD0001; *e*-value 0.0) and the replicative helicase (CD3657; *e*-value 7 × 10^−713^) were identified with high confidence, sharing respectively 62% and 52% identity with their *B. subtilis* counterparts across the full length of the protein. Thus, CD0001 and CD3657 are very likely to correspond to the replication initiator protein and replicative helicase of *C. difficile*, respectively. Interestingly, no homologue of BsDnaB was found using this strategy, indicating the absence of a protein with substantial primary amino acid similarity to DnaB in *C. difficile*. However, BLASTP shows that the genome of *C. difficile* does harbour two homologues of BsDnaD (CD2943: *e*-value = 2 × 10^−5^, identity = 29%, query coverage = 32%; CD3653: *e*-value = 4 × 10^−5^, identity 29%, query coverage 47%). As BsDnaB and BsDnaD are strictly required for replication initiation in *B. subtilis* and are structurally related despite limited amino acid sequence similarity [18], we further examined the *C. difficile* homologues of BsDnaD. BsDnaD is composed of two domains: DDBH1 (DnaD DnaB Homology 1) and DDBH2 (DnaD DnaB Homology 2, also known as PFAM DnaB_2). BsDnaB has a DDBH1-DDBH2-DDBH2 structure [18]. The DnaD-like proteins CD3653 and CD2943 of *C. difficile* both consist of three domains (DDBH1-DDBH2-DDBH2) and therefore resemble BsDnaB in domain structure (figure 1*a*). CD2943 is annotated as a putative phage replication protein and is located in the approximately 50 kb prophage 2 [65]. Also in *Listeria monocytogenes*, *S. aureus* and *Lactobacillus plantarum* DDBH2-containing phage, genes have been identified [18,66]. Considering the fact that the prophage is not part of the *C. difficile* core genome, we consider a role for CD2943 in chromosomal DNA replication unlikely, but a role for CD3653 plausible.

A similar argument can be made for the putative helicase loader. Two homologues of the BsDnaI protein were identified in *C. difficile* by BLASTP (CD3654: *e*-value = 8 × 10^−10^, identity 26%, query coverage = 46%; CD0410: *e*-value = 1 × 10^−18^, identity = 31%, query coverage = 51%). The sequence homology of these putative loader proteins with their counterpart in *B. subtilis* is mainly confined to the C-terminal AAA+ domain that contains the Walker A and B motifs [59]. CD0410 is located on the conjugative transposon CTn*2* [67] and therefore not part of the core genome of *C. difficile*; this makes a role as a chromosomal replication intiation protein unlikely. The *dnaD-*like gene CD3653 is located adjacent to the putative loader (CD3654), in the same genomic region as the replicative helicase (CD3657) (figure 1*b*). Of note, the *dnaB* gene of *B. subtilis* is located next to the gene helicase loader *dnaI* [68], suggesting a functional relationship between the loader ATPase and a DnaB_2 family protein. Indeed, it has been suggested that BsDnaB is a co-loader of the BsDnaC helicase [14]. The genomic region containing CD3654 and CD3657 is conserved and essential [69], and our analyses therefore strongly suggest that CD3654 is the cognate loader protein for the *C. difficile* replicative helicase CD3657.

A BLASTP query identified a single protein homologous to *B. subtilis* primase BsDnaG. This protein, CD1454, shared 31% identity with its *B. subtilis* counterpart across the full length of the protein (*e*-value = 9 × 10^−98^), and contains all domains expected for a bacterial primase (electronic supplementary material, figure S1). Thus, CD1454 probably encodes the primase for chromosomal DNA replication.

### 2.2. Helicase can form hexamers at high concentration

A distinguishing feature of the different modes of helicase loading (ring-maker versus ring-breaker) is the multimeric state of helicase at dilute concentrations of protein [6]. Therefore, we purified recombinant *C. difficile* helicase protein and determined its multimeric state using analytical gel filtration. At concentrations below 5 µM, we observed predominantly monomeric protein, with a fraction of the protein forming low molecular weight (MW) complexes (probably dimers or trimers) while at 10 µM and above, the helicase formed larger oligomers, most probably hexamers (electronic supplementary material, figure S2). Thus, at physiological (nM) concentrations the *C. difficile* helicase is predominantly monomeric, suggesting it is of the ring-maker type, like *B. subtilis*. Multimerization at high concentrations of protein was independent of the presence of ATP (G.S.B., P.S., V.P. & M.G. 2014, unpublished data).

**Figure 2.**
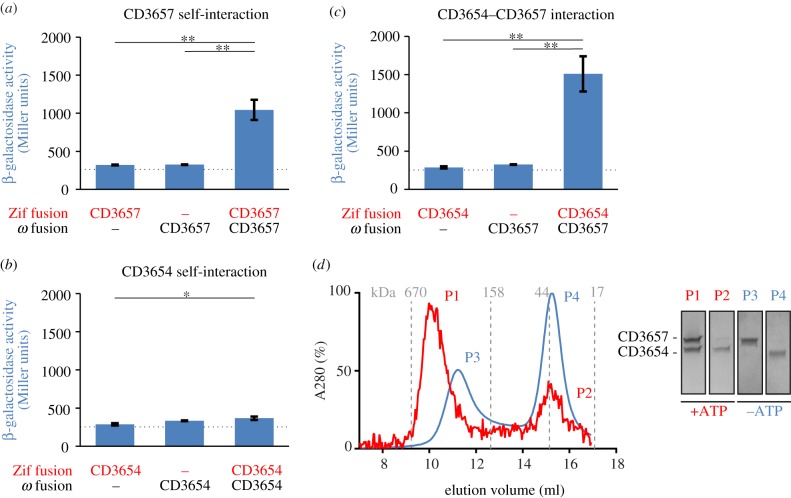
CD3654 and CD3657 interact. (*a*) Bacterial two-hybrid analysis of CD3657 self-interaction. (*b*) Bacterial two-hybrid analysis of CD3654 self-interaction. (*c*) Bacterial two-hybrid analysis of the CD3657–CD3654 interaction. Bar graphs in (*a*–*c*) indicate average values and error bars indicate standard deviation of the measurements (*n* = 3). Significance was determined using the Student's *t*-test (*p < 0.05, **p < 0.001). (*d*) CD3657 and CD3654 interact in an ATP-dependent manner. Analytical gel filtration was performed on a HiLoad 10/300 GL Superdex analytical grade size exclusion column with 2.21 µM (monomer) of CD3657 and CD3654 in the presence (red) and absence (blue) of 1 mM ATP. Inset shows a Coomassie-stained SDS-PAGE gel of the numbered peak fractions. Dashed line indicates the maximum background level of β-galactosidase expression observed in our experimental set-up.

To confirm the gel-filtration data, we investigated the self-interaction of helicase in a bacterial two-hybrid system [70]. This system detects interactions between a protein fused to Zif (Zinc-finger DNA binding domain) and a protein fused to the RNA polymerase *ω* subunit. Interaction between proteins of interest facilitates transcriptional activation of a Zif-dependent *lacZ* reporter gene in a dedicated reporter strain [70]. In order to quantify the interaction, *E. coli* cells containing the plasmids encoding the fusion proteins were permeabilized and assayed for β-galactosidase activity. We found that in the reporter strain transformed with plasmids harbouring both fusion proteins, β-galactosidase activity was approximately threefold higher than for the reporter strains harbouring the individual plasmids, indicating a clear self-interaction for the *C. difficile* helicase protein (figure 2*a*).

Similar experiments were carried out with the putative helicase loader (CD3654). Analytical gel filtration using purified loader protein (CD3654) showed that it was monomeric at all concentrations tested (P.S., V.P. & M.G. 2014, unpublished data). Consistent with this observation, no self-interaction of CD3654 was found in the bacterial two-hybrid system (figure 2*b*).

We conclude that helicase can form homomultimeric assemblies, whereas the putative loader is monomeric under the conditions tested.

### 2.3. Helicase and the putative helicase loader interact in an ATP-dependent manner

If CD3654 is a legitimate loader for the *C. difficile* helicase (CD3657), we expect that the proteins interact *in vivo* and *in vitro*. To determine if this is the case, we performed bacterial two-hybrid and analytical gel-filtration experiments. First, we tested if an interaction between the helicase CD3657 and the putative loader CD3654 could be demonstrated in the bacterial two-hybrid system. CD3657 was fused to the RNA polymerase *ω* subunit and CD3654 was fused to Zif (Karna 2010). The β-galactosidase activity in the reporter strain containing both plasmids was approximately fivefold increased (*p* < 0.001) compared with the reporter strains containing the individual plasmids (background) (figure 2*c*). The highly significant increase in β-galactosidase activity implies substantial interactions between the *C. difficile* helicase and putative helicase loader. Similar results were obtained when CD3657 was fused to Zif and CD3654 to the RNA polymerase ω subunit (E.v.E., A.F. & W.K.S. 2015, unpublished data). This suggests that the combination of protein and fusion domain does not influence the results of this assay.

To exclude the possibility for false negative or false positive results as a result of the two-hybrid system, we additionally performed analytical gel-filtration experiments using purified non-tagged CD3657 and CD3654 proteins. In these experiments, *C. difficile* helicase and loader were combined in equimolar concentrations (2.21 µM of monomer) in the presence and absence of ATP (1 mM). In the presence of ATP, the elution profile showed a major high MW peak (approx. 10 ml; approx. 500 kDa, P1) and a minor low MW peak (approx. 15 ml; approx. 40 kDa, P2) (figure 2*d*). In combination with a visual inspection of the fractions collected from both peaks on an Coomassie-stained SDS-PAGE gel, we believe that the major peak can be attributed to a large complex (most probably a dodecameric assembly consisting of six CD3657 monomers and six CD3654 monomers; theoretical MW 522 kDa), while the minor peak corresponds to predominantly free monomeric CD3654 (theoretical MW 38 kDa). Similar results were obtained when a high concentrations of proteins (approx. 10 µM) were used (electronic supplementary material, figure S3), suggesting that pre-formed hexameric helicase retains the ability to interact with the CD3654 protein at the same stoichiometry.

The elution profile of the same concentration of proteins in the absence of ATP showed a completely different picture (figure 2*d*). A minor peak was observed at approximately 11 ml (approx. 300 kDa, P3) and a major second peak eluted at approximately 15 ml (approx. 40 kDa, P4). MW estimates, in combination with an evaluation of peak fractions on an SDS-PAGE gel, indicated that the first peak most probably corresponds to a complex of six monomers of CD3657 (theoretical MW 297 kDa), while the second peak corresponds to monomeric CD3654 protein.

Together, the data show that the CD3657 helicase and the loader CD3654 can form a complex in an ATP-dependent manner.

### 2.4. Mutation of the helicase Walker A motif abrogates protein–protein interactions

To address the question of which of the proteins (or whether both) require ATP to promote the formation of a CD3657-CD3654 complex, mutants in the Walker A motif of both proteins were created. The Walker A motif (GXXXXGK [T/S]) directly and indirectly interacts with ATP, and is the principal ATP-binding motif of P-loop ATPases [26]. The motif is highly conserved in both helicase and helicase-loader proteins [7]. The lysine residue (K) forms a direct interaction with the negatively charged nucleotide β or γ phosphate group, and mutation of this residue is known to abrogate nucleotide binding and lead to inactivation of P-loop ATPases. The threonine (T) residue in the Walker A motif either directly or indirectly coordinates a Mg^2+^ within the ATP-binding site, which in turn coordinates the phosphate groups of ATP. In the *G. stearothermophilus* replicative helicase, mutation of the threonine residue results in a protein that lacks ATPase and unwinding activities [59]. Based on this knowledge, the equivalent residues were identified in the *C. difficile* helicase protein. Using site-directed mutagenesis, we generated mutant helicase proteins in which the lysine at position 214 was changed into an arginine (K214R) and the threonine at position 215 was changed into an alanine (T215A).

**Figure 3.**
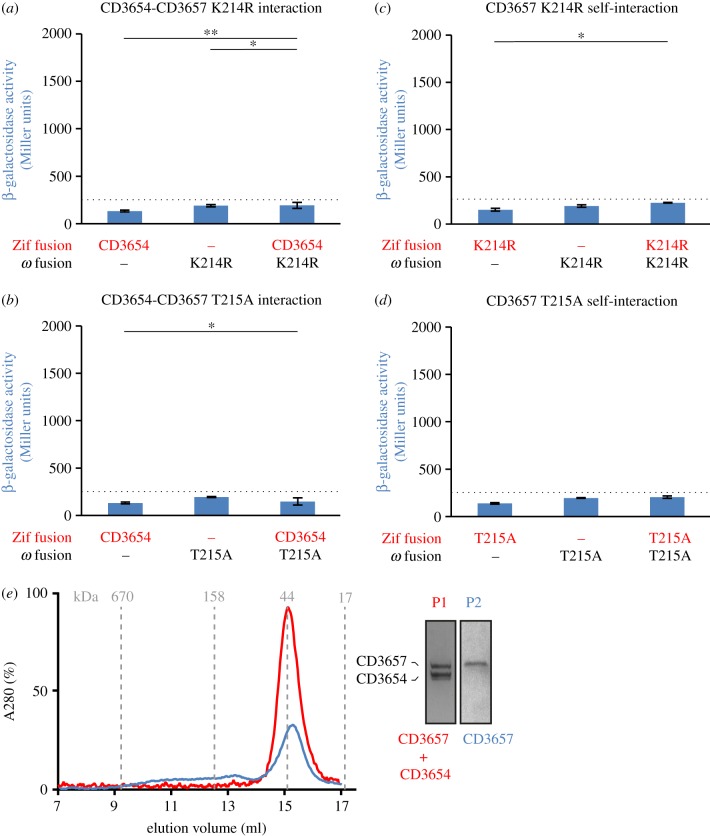
Walker A mutants of CD3657 are defective in protein–protein interactions. (*a*,*b*) Walker A mutants of CD3657 ((*a*) K214R; (*b*) T215A) show no or severely reduced interactions with the putative loader protein CD3654 in a bacterial two-hybrid assay. (*c*,*d*) Walker A mutants of CD3657 ((*c*) K214R; (*d*) T215A) no longer self-interact in a bacterial two-hybrid assay. Bar graphs in (*a*–*d*) indicate average values and error bars indicate standard deviation of the measurements (*n* = 3). Significance was determined using the Student's *t*-test (*p < 0.05, **p < 0.001). (*e*) CD3657 T215A is defective in protein–protein interactions. Analytical gel filtration was performed on a HiLoad 10/300 GL Superdex 200 analytical grade size exclusion column with 2.43 µM of CD3657 T215A in the presence (red) and absence (blue) of 2.43 µM CD3654. Inset shows a Coomassie-stained SDS-PAGE gel of the numbered peak fractions. Dashed line indicates the maximum background level of β-galactosidase expression observed in our experimental set-up.

To determine whether these CD3657 proteins showed altered protein–protein interactions, we performed bacterial two-hybrid experiments. We fused the CD3657 protein to the ω-subunit and CD3654 or CD3657 proteins to the Zif subunit and evaluated their ability to drive the expression of a transcriptional reporter. For both the CD3657 K214R and CD3657 T215A mutants, interaction with the wild-type CD3654 protein were severely reduced or completely lost (figure 3*a*,*b*). This prompted us to investigate if the mutant helicases still had the capacity to form homomultimeric assemblies, as it has been shown in other bacteria that oligomerization is an important step in the mechanism of action of DNA helicases [71]. We found that the CD3657 K214R also demonstrated reduced (possibly absent) self-interactions, whereas the CD3657 T215A mutant had completely lost the ability to self-interact (figure 3*c*,*d*). We conclude that the ability of the CD3657 helicase to coordinate ATP correlates with its ability to interact with the putative loader CD3654 and the ability to self-interact. As the most dramatic effect was observed for CD3657 T215A, we focused our further experiments on this particular mutant.

To confirm the findings from the bacterial two-hybrid experiments, we purified CD3657 T215A protein and subjected it to size exclusion chromatography (figure 3*e*). *Clostridium difficile* helicase T215A mutant (2.43 µM) was incubated in the presence of ATP (1 mM) and loaded onto a size exclusion column. A major peak was observed at approximately 15 ml (approx. 40 kDa), probably corresponding to monomeric CD3657 T215A (theoretical MW 49 kDa). We did not observe any high MW complexes under these conditions, in contrast with the wild-type CD3657 protein (figure 2*d*). Next, we combined the CD3657 T215A mutant and the wild-type CD3654 protein (both 2.43 µM) in the presence of ATP (1 mM) (figure 3*e*). A single peak was observed at approximately 15 ml (approx. 40 kDa). Analysis of the peak fractions on SDS-PAGE demonstrated that the peak contained both CD3657 T215A and CD3654 protein, and thus corresponds to monomeric forms of both proteins (theoretical MW 49 and 38 kDa, respectively). Also in these experiments, no high MW complexes were found, in contrast with the wild-type CD3657 and CD3654 proteins (figure 2*d*).

We also generated constructs with Walker A mutations in CD3654 (K198R, T199A) and tested these in the bacterial two-hybrid assay and in size exclusion chromatography. The mutant loader proteins retained the ability to interact with the wild-type helicase protein (electronic supplementary material, figure S4). Self-interaction was not observed for the mutant loader proteins, concordant with the results obtained with the wild-type loader protein (electronic supplementary material, figure S5).

Overall, our data show that the Walker A mutant CD3657 T215A can no longer self-interact and has lost the capacity to interact with the putative loader protein CD3654. We conclude that the ATP requirement for the interaction between the two proteins is most likely to be the result of ATP binding to CD3657.

### 2.5. Helicase loading of *Clostridium difficile* differs from *Bacillus subtilis*

So far, our data show that the replicative helicase of *C. difficile*, CD3657, interacts in an ATP-dependent manner with the helicase-loader protein CD3654 and that loading probably occurs via a ring-maker mechanism, as for *B. subtilis*. In *B. subtilis*, stimulation of activity of the (monomeric) helicase protein by the loader protein was clearly shown using an *in vitro* helicase activity assay [17,57]. Therefore, we set out to investigate if the activity of *C. difficile* helicase could be reconstituted in the presence of the CD3654 protein in a similar experiment. DNA-unwinding helicase activity was assayed by monitoring and quantifying the displacement of a radiolabelled oligonucleotide annealed to single-stranded circular M13mp18 DNA. To enable loading of helicase, the 5′ end of the oligonucleotide contained a poly(dCA) tail that produces a forked substrate upon annealing of the complementary region to ssM13. Wild-type CD3657 and CD3654 proteins were mixed in equimolar concentrations (monomers) in the presence of ATP and reaction buffer, and displacement of the radiolabelled oligonucleotide was monitored over time. In contrast with the *B. subtilis* proteins, helicase activity was not observed during this time course (electronic supplementary material, figure S6). This suggests that another factor is required for *in vitro* loading and/or activation of the *C. difficile* helicase.

### 2.6. *Clostridium difficile* helicase is activated by primase

Primase has been shown to interact with helicase and stimulate its activity in a variety of organisms [16,57,61]. Therefore, we wanted to investigate if *C. difficile* helicase could be activated by primase. We purified the *C. difficile* primase without an affinity tag to more than 95% purity. SEC-MALS/dRI analysis showed a single peak corresponding to a MW of 74 kDa, indicating that the primase is monomeric under the conditions tested (expected MW 70 kDa) (electronic supplementary material, figure S7). We performed helicase assays to determine the effect of primase on helicase activity.

**Figure 4.**
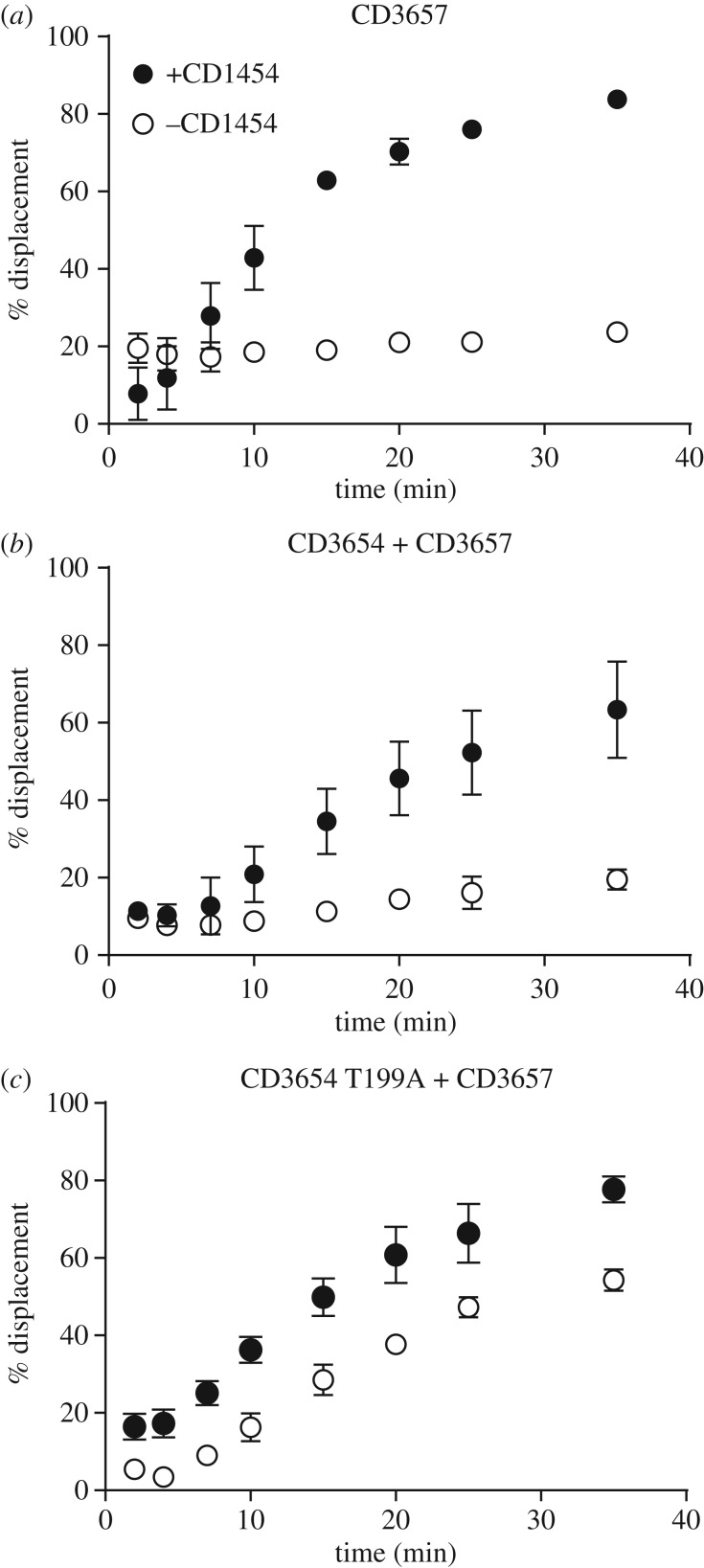
Helicase activity is stimulated by primase. Helicase activity was assayed by quantifying the displacement of a radiolabelled (γ^32^P-ATP) oligonucleotide partially annealed to the single stranded circular DNA m13mp18. Per cent displaced signal from the helicase assays in time. (*a*) Helicase activity of the CD3657 helicase with and without the CD1454 primase. (*b*) Helicase activity of the CD3657 helicase in the presence of the putative loader protein CD3654, in the presence and absence of the CD1454 primase. (*c*) Helicase activity of the CD3657 helicase in the presence of a representative Walker A mutant (T199A) of the putative loader protein CD3654, in the presence and absence of the CD1454 primase. The other mutants of CD3654 (K198R, D258Q) gave similar results (electronic supplementary material, figure S7) but have been omitted for clarity. Error bars indicate standard deviation (*n* = 3).

First, we determined if the CD3657 helicase displayed activity in this particular assay in the absence of any other proteins. Approximately 20% of the oligonucleotide was displaced after as little as 2 min of incubation, and this fraction remained stable over the course of 35 min (figure 4*a*). This suggests that helicase may be capable of self-loading but displays marginal DNA-unwinding activity by itself.

When the CD1454 primase and CD3657 helicase were combined in equimolar amounts of monomer, a 3.5-fold increase in displacement of the oligonucleotide (up to approx. 80%) was observed after 35 min (figure 4*a*). This indicates that primase has a profound stimulatory effect on helicase activity in this assay. Strikingly, strand displacement by helicase seems inhibited in the presence of primase compared with helicase alone at early time points (less than 10 min). This suggests that primase may inhibit self-loading, in addition to its role as an activator of helicase activity. Control experiments using only primase did not result in significant displacement of the oligonucleotide, demonstrating that the displacement is not the result of an inherent property of the primase protein (P.S., V.P. & M.G. 2015, unpublished data).

The results from the helicase assay suggest a functional interaction between the helicase and primase proteins. We tried to validate the interaction using a bacterial two-hybrid system. Despite our efforts, no interaction could be demonstrated between the full-length primase and helicase proteins, or the helicase-interacting domain of primase and helicase in bacterial two-hybrid experiments (E.v.E., A.F. & W.K.S. 2015, unpublished data). This suggests that the interaction between primase and helicase may be very weak (below detection limit of the assay) and/or transient, concordant with observations in *E. coli* [47], or that the bacterial two-hybrid system does not allow recapitulation of the conditions necessary for the interaction between the helicase and primase proteins.

### 2.7. The putative loader protein CD3654 ‘locks’ the CD3657 helicase

Next, we set out to investigate the role of the putative loader CD3654 on the helicase activity of CD3657 in the presence and absence of primase. We observed a very low level of displacement (less than 20%) in our helicase assay in the presence of both helicase and the putative loader (figure 4*b*). Notably, within the first 10 min of the assay the fraction of displaced oligonucleotide did not exceed 10%, in contrast with the situation with helicase alone where it reached 20% (figure 4*a*). At endpoint, the fraction was comparable between the two conditions. This suggests that—at least at early time points—the presence of the putative loader negatively affects helicase activity.

We wondered if the positive effect of the CD1454 primase on CD3657 helicase activity could be observed in the presence of both the putative loader ATPase CD3654 and helicase. From 8 min on, a clear stimulation of helicase activity by primase was observed, reaching approximately 60% displacement over a time course of 35 min (figure 4*b*). Interestingly, the addition of the putative loader protein resulted in a 20% reduction in strand displacement over a time course of 35 min compared with results obtained with a combination of helicase and primase (approx. 80%; figure 4*a*). We conclude that primase can activate helicase activity in the presence of the putative loader, but that the loader retains a negative effect on overall helicase activity in this assay.

To exclude the possibility that the displacement observed in our helicase assays could be attributed to another protein than the CD3657 helicase, we used the CD3657 K214R and T215A mutants. We observed only background levels (less than 5%) of displacement in the presence of CD3657 mutant helicases, primase and the putative loader (electronic supplementary material, figure S8) in comparison with approximately 60% strand displacement for the wild-type helicase in the presence of the same proteins (figure 4*b*). This shows that the displacement observed in our experiments can be attributed to helicase alone and not some other factor.

In *E. coli*, interaction between the loader ATPase protein and helicase does not require ATP binding by the loader [8,72,73]. However, hydrolysis of ATP to ADP by the loader (which results in dissociation from the helicase-loader complex) appears crucial to lift the negative effect of the loader on helicase activity [8,72,73]. Therefore, we set out to investigate the effect of CD3654 proteins with a mutated Walker A or Walker B motif on helicase activity. The lysine residue in (K198) in the Walker A motif is predicted to directly interact with ATP, and mutation of homologous residues is known to eliminate appropriate ATP binding of AAA+ proteins, resulting in inactivation [26]. The threonine residue in Walker A (T199) and the aspartic acid residue in Walker B (D258Q) are predicted to be involved in coordination of an Mg^2+^ ion within the ATP-binding site. Overall, the three mutants (K198R, T199A and D258Q) should be affected in their ability to coordinate and/or hydrolyse ATP.

We found a 2.5-fold higher displacement (approx. 50%) of the oligonucleotide in the helicase assays with all three CD3654 Walker A and B mutants (figure 4*c*; electronic supplementary material, figure S9) compared with the wild-type (figure 4*b*) in the absence of primase. Interestingly, helicase activity in assays combining wild-type CD3657 helicase, CD1454 primase and mutants of the putative loader was similar (approx. 80%; figure 4*c*) to the activity measured in the two protein helicase–primase experiment (figure 4*a*). This suggests that binding and/or hydrolysis of ATP by the putative loader is not required to deliver the CD3657 helicase to the DNA, but is at least partially responsible for the negative effect (‘locking’) of the helicase activity.

### 2.8. *Clostridium difficile* primase trinucleotide specificity is similar to *Aquifex aeolicus* primase

**Figure 5.**
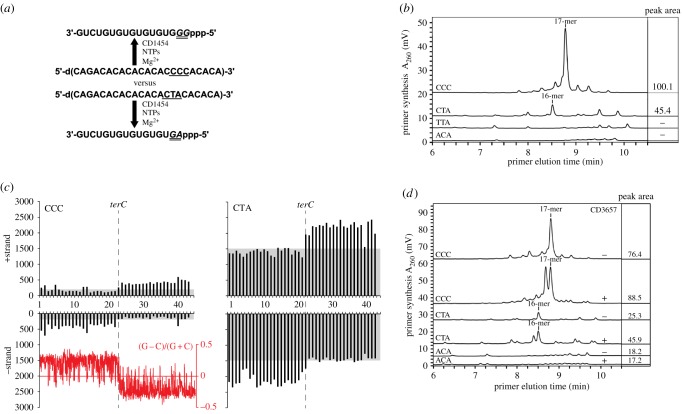
CD3657 affects trinucleotide specificity of CD1454. (*a*) Schematic depiction of the primase activity assay using two different single-stranded DNA templates, along with the resulting full-length RNA primer. (*b*) Analysis of CD1454 priming activity at different trinucleotides. (*c*) The number of CCC and CTA trinucleotides on the + and − strand of the *C. difficile* 630Δ*erm* was calculated in 100 000 bp bins. Skew in trinucleotide frequency is highlighted using a grey box. The position of the putative terminus of replication (*terC*) is indicated with a vertical dashed line. The red inset shows the GC skew, as calculated in Artemis [75] with a window size of 5000. (*d*) CD3657 affects CD1454 primase activity. The amount of primer produced by CD1454 in the presence (+) or absence (−) of CD3657 on ssDNA templates with different trinucleotides at stoichiometric concentrations of [CD3657]_6_:[CD1454]_3_. The numbers in the panel to the right of the chromatograms in (*b*) and (*d*) denote the total peak area for the RNA primer products synthesized and shown in the associated chromatogram to the left.

Above, we have established a crucial role for the *C. difficile* primase CD1454 in the activation of the CD3657 helicase, which has not been reported for Gram-positive bacteria before. Next, we sought to evaluate primase activity. To confirm enzymatic activity and identify the template initiation motif, two 50-mer oligonucleotides that comprised all 64 potential trinucleotide sequences were used in a priming assay, as previously described [74]. These results confirmed enzymatic activity and revealed likely trinucleotide motifs that supported de novo primer synthesis (M.A.L. 2014, unpublished data). Next, a more detailed analysis of template specificity was performed using a 23-mer ssDNA-template containing the trinucleotide of interest (figure 5*a*). We found that the CD1454 primase preferentially initiated de novo RNA primer synthesis on the initial 5′-d(CCC) motif encountered by this enzyme within the 23-mer ssDNA template; therefore, a 17-mer rather than an anticipated 16-mer RNA primer was produced (figure 5*b*). The 5′-d(ACA)-specific 23-mer ssDNA was tested in the priming assay and no RNA primers were synthesized by *C. difficile* primase, further demonstrating a requirement for the appropriate template initiation motif (figure 5*b*). The activity of the CD1454 was notably inhibited by Mg^2+^ concentrations more than 30 mM, and largely independent of NTP concentration (more than 1 mM) under the conditions of the assay (electronic supplementary material, figure S10).

The observed template specificity for the CD1454 primase differs from most other bacterial primases evaluated to date. More specifically, 5′-d(CTA) only supported minimal priming and no initiation occurred on 5′-d(TTA) by the *C. difficile* primase (figure 5*b*), whereas substantial dinucleotide polymerization occurred on these two trinucleotides by primases from other Firmicutes such as *G. stearothermophilus*, *S. aureus* and *Bacillus anthracis* [44,53,76]. The trinucleotide 5′-d(CTG) was also not an effective template for *C. difficile* primase (M.A.L. 2014, unpublished data), whereas it is efficiently primed by primase from Gammaproteobacteria such as *E. coli*, *Pseudomonas aeruginosa* and *Yersinia pestis* [76–78]. To date, only the primase from the hyperthermophile *Aquifex aeolicus* has been shown to initiate primer synthesis on the trinucleotide 5′-d(CCC) [74].

As primase presumably scans the ssDNA template in the 3′ to 5′ direction until an initiation trinucleotide is encountered, the influence of the 5′ nucleotide adjacent to the preferred 5′-d(CCC) trinucleotide motif was evaluated. We found that CD1454 primase was most efficient at initiating de novo primer synthesis when the 5′ nucleotide was a cytosine or—with a slightly lower efficiency—a thymine (electronic supplementary material, figure S11). Considerably less priming occurred when the 5′ adjacent nucleotide contained an adenine base and only marginal non-specific priming occurred when this nucleotide contained a guanine (electronic supplementary material, figure S11). These results suggest that the nucleotide 5′ to the preferred trinucleotide influences the catalytic activity at the active site for subsequent initiation of de novo primer synthesis.

### 2.9. Helicase influences trinucleotide specificity of primase

The identification of the 5′d(CCC) motif as the preferred trinucleotide for initiation by the CD1454 primase was unexpected, as the *C. difficile* chromosome has a G + C content of only 29% [65], and other low-GC Firmicutes, such as *S. aureus* and *G. stearothermophilus* [44,53], preferentially initiate at 5′d(CTA). Therefore, we evaluated the relative frequency of the CCC motif on the plus and minus strand of the *C. difficile* chromosome and compared it with the relative frequency of CTA motif on which the CD1454 initiates less efficiently (figure 5*c*). Our analysis showed that CTA trinucleotides were on average fivefold to 10-fold more frequent within the *C. difficile* chromosome than the preferred CCC motif. Strikingly, there appears to be a strand bias in the occurrence of the motifs, which mirrors the GC-skew ([G − C]/[G + C]) of the chromosome (figure 5*c*). This suggests that the motifs are preferentially associated with the lagging strand where primase acts and indicates a possible role for primase in generating the strand bias.

As it has previously been shown that helicase can stimulate primase activity, affect primer length and modulate trinucleotide specificity in *E. coli* [48,49,51], we determined whether the CD3657 helicase could enhance priming at the non-preferred trinucleotide. The effect of CD3657 helicase on CD1454 primase activity was evaluated using 23-mer ssDNA templates that contained the preferred trinucleotide 5′-d(CCC), the Firmicute-preferred trinucleotide 5′-d(CTA) or 5′-d(ACA) (negative control). RNA primer production by the primase CD1454 was strongly stimulated on 5′-d(CTA) in the presence of the CD3657 helicase (45.9 versus 25.3 nMole) at stoichiometric concentrations of [CD3657]_6_:[CD1454]_3_. Helicase-stimulated primase activity on 5′-d(CCC) increased RNA primer synthesis by only 1.15-fold and, as expected, no stimulation of RNA primer synthesis occurred on the 5′-d(ACA) trinucleotide (figure 5*d*). We also observed that helicase stimulated primase to synthesize slightly shorter RNA polymers (figure 5*d*). The production of a shorter primer might allow faster transfer to the replicative polymerase, increasing the replication speed. Collectively, our data show that the stimulatory effect of helicase on primase activity *in vitro* is clearly enhanced on the trinucleotide 5′-d(CTA), which is preferred by other Firmicute primases studied to date, whereas the effect at the *C. difficile*-preferred 5′-d(CCC) sequence was only minimal, probably due to the already high efficiency of priming on this motif by the CD1454 primase.

We conclude that through the interaction of primase with helicase and the relative abundance of the 5′d(CTA) the overall efficiency of priming is likely to be greatly enhanced.

### 2.10. A lysine residue contributes to trinucleotide specificity of primase

Considering that the *C. difficile* primase trinucleotide specificity (figure 5*b*) is unusual for Firmicutes, but resembles that of the hyperthermophilic *A. aeolicus* primase [74], we wanted to determine the factors that might contribute to this trinucleotide template specificity for subsequent dinucleotide polymerization.

**Figure 6.**
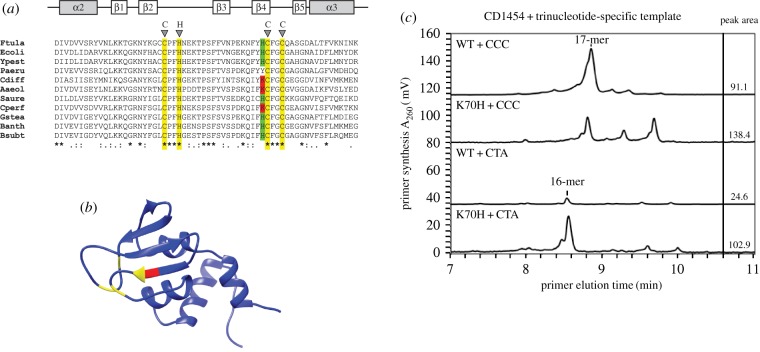
A lysine residue affect trinucleotide specificity of CD1454. (*a*) Alignment of the α2–α3 region of the zinc-binding domain of primases with characterized trinucleotide specificity. Predicted secondary structure (α helix in grey, β sheet in white) is indicated above the alignment. Ftula, *Francisella tularensis;* Ecoli, *Escherichia coli*; Ypest, *Yersinia pestis*; Paeru, *Pseudomonas aeruginosa*; Cdiff, *Clostridium difficile*; Aaeol, *Aquifex aeolicus*; Saure, *Staphylococcus aureus*; Cperf, *Clostridium perfringens*; Gstea, *Geobacillus stearothermophilus*; Banth, *Bacillus anthracis*; Bsubt, *Bacillus subtilis*. Highlighted are the zinc coordinating residues of the CHC2 zinc-binding motif (yellow), and the adjacent histidine (green) or lysine (red) residues. Sequence conservation is indicated below the alignment. (*b*) Ribbon representation of the CD1454 primase zinc-binding domain indicating the zinc coordinating residues (yellow) and the location of the lysine residue (red). (*c*) Thermally denaturing high-performance liquid chromatography analysis of primase activity of wild-type (WT) and K70H mutant CD1454 primase protein with the CCC and CTA containing templates. The primase (WT or K70H mutant) and initiation trinucleotide in the single-stranded 23-mer template used is shown in the upper left corner of the respective chromatogram. The numbers in the panel to the right of the chromatograms denote the total peak area for the RNA primer products synthesized and shown in the associated chromatogram to the left. The mutation affects RNA primer synthesis from both preferred 5′-d(CCC) and non-preferred 5′-d(CTA) trinucleotides but shows the strongest increase in activity from the 5′-d(CTA) trinucleotide.

We performed pair-wise sequence comparisons between the ZBD that is involved in sequence specific DNA binding of primase in *C. difficile* and 10 other bacterial species (*Clostridium perfringens*, *G. stearothermophilus*, *S. aureus*, *B. anthracis*, *B. subtilis*, *E. coli*, *P. aeruginosa*, *Y. pestis*, *A. aeolicus* and *Francisella tularensis*). *Clostridium perfringens* (53.8% identity and 87.9% similarity in 91 residues) had the highest amino acid sequence homology, followed by *B. anthracis* (49.5% identity and 87.9% similarity in 91 residues) and *A. aeolicus* (47.2% identity and 83.1% similarity in 89 amino acids). The *E. coli* ZBD had the lowest homology (43.2% identity and 83.0% similarity in 88 residues). The Clustal Omega alignment tool (http://www.ebi.ac.uk/Tools/msa/clustalo) placed the *C. difficile* CD1454 ZBD closest to the ZBD of *A. aeolicus* and *G. stearothermophilus* (figure 6*a*). The (partial) clustering of the *C. difficile* CD1454 primase with other bacterial primases that have different trinucleotide specificity led us to anticipate that the composition and spatial location of specific amino acids in the ZBD relative to the RNA polymerase domain in primase must be critical for template recognition and phosphodiester bond formation.

In the multiple sequence alignment, a lysine (K) at residue 70 in the ZBD of *C. difficile* primase was unique to *C. difficile*, *C. perfringens* and *A. aeolicus* (figure 6*a*). Structural modelling of CD1454 with Phyre2 [79] revealed that this particular lysine residue is in close proximity to the zinc-ribbon motif that tetrahedrally coordinates a zinc ion and is essential for primase function (figure 6*b*; electronic supplementary material, figure S1) [80]. Interestingly, the PDB template used by Phyre2 is 2AU3, the *A. aeolicus* primase [54]. The crystal structure of *G. stearothermophilus* DnaG primase contains a histidine at this position [50,80] and the multiple sequence alignment suggests that other Firmicutes also do. We hypothesized that the exposed lysine residue influences primase initiation specificity. To address this experimentally, we mutated the K70 residue of the CD1454 primase to a histidine. If the lysine contributes to the unusual trinucleotide specificity of the *C. difficile* primase, we would expect reduced priming on the 5′-d(CCC) motif by the CD1454 K70H mutant compared with the wild-type CD1454 primase. Indeed, CD1454 K70H showed substantially reduced initiation on the 5′-d(CCC) motif and enhanced priming on the 5′d(CTA) motif (figure 6*c*). The CD1454 K70H synthesized slightly more primers than wild-type primase due to relaxed template specificity, as evidenced by the synthesis of RNA polymers longer than 17 nucleotides in length (figure 6*c*).

Importantly, these results demonstrate that modifying a single residue within the ZBD of the CD1454 primase is sufficient to alter initiation specificity and suggests that the exposed lysine residue is crucial for preferential initiation on the 5′-d(CCC) motif.

## 3. Discussion

*In silico* analysis of the *C. difficile* genome failed to identify a homologue of DnaB of *B. subtilis*, which is generally considered the model for Gram-positive bacteria. BsDnaB, together with BsDnaD and BsDnaI, is strictly required for helicase loading in *B. subtilis in vivo* [12–14]. A homologue of BsDnaD was identified that may be involved in DNA replication (CD3653; *e*-value 4 × 10^−5^), although query coverage (47%) and identity (29%) were low. This situation is reminiscent of that in some Mollicutes, where also only a *DnaD-*like gene was identified [15]. Despite a lack of clear homology at the primary amino acid sequence level, BsDnaB and BsDnaD are structural homologues [18]. Fusions of these proteins, are found in phage-related replication proteins and it was suggested that in the absence of DnaB, a single fusion protein may couple or combine both functions [15,66]. Nevertheless, the situation in *C. difficile* differs from those in phage and Mollicutes. Structure predictions reveal that the phage-related and the Mollicutes DnaD-like proteins have a two-domain structure containing one copy of the DDBH1 and DDBH2 domain [18], and the proposed hybrid function of phage proteins is based on limited local amino acid sequence similarity in the DDBH2 domain only [66]. CD3653 on the other hand has a three-domain structure with a single DDBH1 and two DDBH2 domains, like DnaB, despite the lack of clear sequence similarity to this protein (figure 1). It is tempting to speculate that CD3653 in *C. difficile* may perform functions similar to both DnaD and DnaB in *B. subtilis*, which include origin remodelling and contributing to the helicase loading process [14,15,81]. DDBH2 domains are characterized by an YxxxIxxxW motif [18]. In BsDnaB, this motif is degenerate in the first DDBH2 domain. By contrast, the motif is readily identified in both DDBH2 domains of CD3653 (figure 1). Our data are consistent with a model where an ancestral three-domain DnaD-like protein was duplicated and subsequently diverged in certain Firmicutes like *B. subtilis*.

DnaB-like helicases (note that this nomenclature is based on the *E. coli* protein name) belong to the superfamily IV of DNA helicases, and the functional unit of this protein is a hexamer [26–28,34]. In *E. coli*, the helicase is found to be a stable hexamer over a broad protein concentration range of 0.1–10 µM [82] and is active as a pre-formed multimer. Helicases belonging to the ring-maker class, such as BsDnaC of *B. subtilis*, can occur in a low oligomeric or monomeric state under dilute conditions [6,14]. Our experiments indicated that CD3657 is monomeric in the low micromolar or nanomolar range (electronic supplementary material, figure S2), which is likely to be reflective of the intracellular concentration of protein [72]. *Clostridium difficile* CD3657 can form hexameric assemblies at higher concentrations (electronic supplementary material, figures S2 and S3), but these pre-formed hexamers are inactive, even in the presence of primase (G.S.B. & P.S. 2012, unpublished data), in contrast with the situation in *E. coli*. Our data are therefore consistent with the notion that CD3657 belongs to the ring-maker class of helicases [6].

The interaction between putative helicase and loader protein was found to be ATP dependent (figures 2 and 3). Mutations in the conserved Walker A and B motifs of the putative loader protein did not fully abrogate the interaction with the wild-type helicase (electronic supplementary material, figure S4). Similarly, in *E. coli*, nucleotide binding to the helicase loader was not a pre-requisite for association with helicase [8,72,83]. Instead, our data indicate that the association of ATP with helicase is crucial for the interaction with the loader protein (figure 3). Notably, there is a correlation between the ability of the helicase to interact with the loader protein and form homohexamers, because a T215A (Walker A) mutant of helicase is defective for both, at least under dilute concentrations of helicase (figure 3). By contrast, the equivalent mutation in *G. stearothermophilus* helicase (T217A) does not affect its ability to form hexamers [59], and the interaction of this protein with *B. subtilis* DnaI readily occurs in the absence of ATP [84].

We attribute the effect on protein–protein interactions of the Walker A mutants of CD3657 to defects in ATP binding rather than hydrolysis (figure 3); a Walker B mutant (CD3657 D318A), but not a mutant that is predicted to be able to bind but not hydrolyse ATP (CD3657 E239A), mirrors our findings with the Walker A mutants in a bacterial two-hybrid assay (E.v.E., A.H.F. & W.K.S. 2015, unpublished data). Binding of the nucleotide to helicase is associated with conformational changes; the N-terminal collar domain constricts upon nucleotide binding in *A. aeolicus* and to a lesser extent *E. coli.* This constricted conformation is believed to favour an interaction with the loader protein [85] and it is therefore conceivable that in the absence of ATP, the *C. difficile* helicase adopts a (dilated) conformation that is incompatible with a functional interaction with the putative loader protein.

Despite substantial bioinformatic and biochemical evidence that CD3654 is indeed the *C. difficile* helicase loader, we found an inhibitory effect of CD3654 on the activity of CD3657 *in vitro*, at least in the presence of CD1454 (figure 4). At first, the negative effect of CD3654 seems at odds with its proposed role as loader. However, in *E. coli* ATP-bound EcDnaC loader protein can act as an inhibitor of the EcDnaB helicase [8,72,73]. We also find that mutants of CD3654 appear capable of loading and/or activating the helicase, but are defective in restraining the DNA-unwinding activity of the helicase like wild-type CD3654 (figure 4*c*; electronic supplementary material, figure S9). Moreover, a functional role for CD3654 in the essential process of helicase loading and/or activation is supported by the observation that no transposon insertions were obtained in the homologue of *cd3654* in an epidemic strain of *C. difficile* (R20291_3513) [69].

Our data strongly suggest that the presence of loader protein alone is not sufficient to load and activate the helicase of *C. difficile*, and that at least one other factor is needed to reconstitute its activity. Indeed, primase is required as an activator of helicase activity in the presence of the loader (figure 4).

Helicases are complex proteins, and their properties can both alter and be altered by other replication factors [85]. DnaB-like helicases consist of two-tiered homo-hexameric rings, one assembled from six subunits of the C-terminal domain and the other formed by the N-terminal domains. The helicase loader interacts with the C-terminal ATPase domain [62,72,86], and the same domain is required for the interaction with the τ subunit of the clamp loader in *E. coli* [87] and *B. subtilis* [88,89]. Strikingly, BsDnaC T217A Walker A mutant fails to form a complex with τ [89]. This finding is very similar to our observations for the interaction between the *C. difficile* helicase and loader.

The N-terminal domain of helicase forms a platform for the interaction with primase in both *E. coli* and *G. stearothermophilus* [62,85]. Unlike the helicase loader, binding of primase to helicase is promoted by a dilated conformation of the N-terminal domain that exposes the interaction surface [58,85]. The helicase–primase interaction is mutually stimulatory, with distinct but overlapping networks of residues in helicase responsible for the modulation of either helicase or primase activity [44,90,91]. Primase binding counteracts the binding of the loader protein in *E. coli* [61], and the loader from *B. subtilis* was found to dissociate from the complex when primase and polymerase bind to helicase [57]. We find that CD1454 stimulates CD3657 helicase activity, independent from the loader (figure 4).

Loading of the CD3657 helicase differs from the situation in other Gram-positive bacteria. Most notably, CD3657 seems to be capable of self-loading, has increased helicase activity in the presence of primase (CD1454) and is negatively influenced by the presence of the putative loader protein (figure 2*a*). *Geobacillus stearothermophilus* helicase demonstrates significant helicase activity by itself [59], and the *B. subtilis* helicase is strongly activated by its cognate loader [17,57]. Instead, helicase loading in *C. difficile* is somewhat reminiscent of the situation in the Gram-negative *H. pylori*, where a dodecameric self-loading helicase remains inactive until activated by primase, leading to the dissociation of the dodecamer into two hexamers [16]. However, we did not find any evidence for dodecameric assemblies for CD3657.

Helicase loading in *C. difficile* also seems to differ in crucial aspects from the Gram-negative model *E. coli*. Walker A mutants of the EcDnaC loader protein are capable of loading the EcDnaB helicase but do not sustain helicase activity, suggesting that ATP turnover by the loader is required to release the helicase [8]. Our data show that in *C. difficile*, (self) loading of CD3657 is stimulated by Walker A mutants of CD3654, but that these mutants of the loader readily release active helicase (figure 2*c*). This suggests a role for ATP binding and/or hydrolysis in ‘locking’ helicase activity.

Further, EcDnaB helicase is in complex with the EcDnaC loader protein throughout the loading process and remains inactive until the EcDnaG primase binds to helicase, thereby releasing the loader protein [61]. Our data do not exclude a similar role for primase in helicase loading of *C. difficile*, but do show that the role of the CD1454 primase is not limited to the release of the putative loader; very strong stimulation of CD3657 helicase activity by CD1454 is observed in the absence of CD3654 (figure 4*a*). We consider two possible (not mutually exclusive) scenarios to explain the activation of helicase by primase in the absence of the loader. First, primase may stabilize the hexameric helicase on the DNA, which indirectly contributes to the unwinding activity. Second, primase may act as a direct activator of the DNA-unwinding activity of helicase. Our experiments do not discriminate between these possibilities, but stabilization of the hexamer or other conformational changes in the hexameric helicase induced by primase have also been observed in *E. coli* [41,58,60,61].

We found that the primase of *C. difficile* has an unusual trinucleotide specificity, with a preference for 5′-d(CCC) (figure 5), which is similar to primase of *A. aeolicus* [74] but different from the specificity of primases from other Firmicutes or Gammaproteobacteria (5′-d(CTA), 5′-d(TTA), 5′-d(CTG)) [50]. The first two nucleotides in all of these trinucleotides are pyrimidines (C or T) and as a result the first two nucleotides in the RNA primer will be purines (G or A). The levels of both ATP and GTP directly or indirectly provide a means by which bacteria can sense the energy status of the cell [92–95]. The nucleotide preference might couple the efficiency of lagging strand DNA synthesis and the nutritional status of the cell.

Priming by the primase CD1454 was highest when the nucleotide 5′ adjacent to the preferred 5′-d(CCC) trinucleotide was a pyrimidine (electronic supplementary material, figure S11), consistent with a previously hypothesized context-dependent enzyme activity [74]. Our results indicate that pyrimidines, probably in part due to the smaller size compared with purines, provide the optimal context for catalysis and dinucleotide polymerization.

We probed the origin of primase trinucleotide specificity and found that a lysine (K) adjacent to the zinc-ribbon motif of CD1454 is important for the optimal physico-chemical environment for primer initiation on 5′-d(CCC) (figure 6). Based on this finding, we would predict a similar specificity for the primase of *C. perfringens*, but our attempts to obtain priming activity with the CPF_2265 primase from *C. perfringens* ATCC133124 have failed thus far.

A 5′-d(CCC) trinucleotide specificity has previously only been observed for *A. aeolicus* [74]. Despite the predicted structural homology between the *A. aeolicus* and *C. difficile* primases (figure 6*b*), this was unexpected, as *C. difficile* is a mesophilic spore-forming Gram-positive pathogen, whereas *A. aeolicus* is a hyperthermophilic Gram-negative bacterium. However, cladistic studies using multiple signature proteins indicate that the *Aquifex* lineage emerged from Gram-positive bacteria, prior to the split of Gram-positive and Gram-negative bacteria [96,97]. These studies concluded that the Firmicutes are presumably among the most ancient bacteria and that the Aquificales have diverged much later in evolution [96]. Indeed, an analysis of the GC content of rRNA clusters suggests that hyperthermophilic species have evolved from mesophilic organisms via adaptation to high temperature [98] and that the Gram-negative double membrane may have been derived from sporulating Gram-positives [99,100]. Thus, the study of *C. difficile* may also contribute to a better understanding of the evolution of the bacterial DNA replication machinery and facilitate the development of antibiotics that target these essential proteins in pathogenic bacteria.

## 4. Material and methods

### 4.1. Plasmid construction and site-directed mutagenesis

All oligonucleotides and plasmids constructed for this study are listed in electronic supplementary material, tables S1 and S2.

To construct the CD3654, CD3657 and CD1454 expression plasmids, the open reading frames were cloned into suitable pET vectors (Novagen) to yield pEVE24, pEVE87 and pEVE7, respectively.

Construction of the plasmids for the bacterial two-hybrid system was performed with Gateway cloning technology (Invitrogen). To construct the CD3654 and CD3657 entry plasmids, the CD3654 and CD3657 open reading frames were introduced into donor vector pDonR™201. Bacterial two-hybrid constructs were made by sub-cloning the genes of interest from the entry plasmids into the destination plasmids pKEK1286 (Zif fusion plasmid) or pKEK1287 (*ω* fusion plasmid) in an LR reaction [70], to yield pEVE118, pEVE120, pEVE122, pEVE123, pEVE124 and pEVE125.

Mutations in plasmids carrying CD3654, CD3654 and CD1454 mutants were constructed according to the QuikChange protocol (Stratagene). Primers were generated with Primer X, a web-based tool for automatic design of mutagenic primers for site-directed mutagenesis.

All constructs were verified by DNA sequencing.

### 4.2. Purification of proteins

Overexpression of all proteins was carried out in *E. coli* BL21 (DE3) from the pET-based vectors pEVE12, pEVE90, pEVE92, pEVE24, pEVE59, pEVE60, pEVE203, pEVE7 and pEVE201 (electronic supplementary material, appendix table S2). Detailed description of the protein purifications is available as the electronic supplementary material. Below, we provide a short summary of the purification process.

Hexameric CD3657 was purified on a Q sepharose column, a heparin sepharose column and a Hiload 26/60 Superdex 200 gel-filtration column. Monomers were recovered after addition of guanidinium chloride solution in incremental steps up to 2M, followed by separation of monomers and multimers on a Hiload 26/60 Superdex 200 column and stored in TED50G20 buffer (Tris pH 7.5 50 mM, EDTA 1 mM, DTT 1 mM, NaCl 50 mM, 20% v/v glycerol) at −80°C after extensive dialysis.

CD3654 was purified on from the flow-through of a Q sepharose and 5 ml SP sepharose columns connected in series, followed by a heparin sepharose and a Hiload 26/60 Superdex 200 gel-filtration column. Purified CD3654 was stored in TED50G10 buffer (Tris pH 7.5 50 mM, EDTA 1 mM, DTT 1 mM, NaCl 50 mM, 10% v/v glycerol) at −80°C.

CD1454 was purified on HiTrap Q HP column in series with a 5 ml heparin sepharose column, followed by a MonoS column and a Hiload 26/60 Superdex 200 column. Protein was stored in TED100G buffer (50 mM Tris pH 7.5, 1 mM EDTA, 1 mM DTT, 100 mM NaCl, 10% v/v glycerol) at −80°C.

Proteins were quantified by UV spectrophotometry and stored at −80°C. Protein purity (more than 95%) was estimated by SDS-PAGE electrophoresis and concentration was determined spectrophotometrically using extinction coefficients calculated by the ExPASy ProtParam tool (http://web.expasy.org/protparam).

Protein concentrations mentioned in this manuscript refer to concentration of the monomer of the protein.

### 4.3. Gel-filtration experiments

Purified proteins were studied in the presence and absence of 1 mM ATP on a Hiload 10/300 GL Superdex 200 analytical grade size exclusion column at a flow rate of 0.5 ml min^−1^. The elution profiles from each experiment were monitored at 280 nm and plotted as a function of the elution volume. To assess interactions between CD3657 and CD3654, purified proteins were mixed in a 1 : 1 stoichiometry (based on monomer concentrations). Samples from fractions were analysed by SDS-PAGE and Coomassie Blue staining to verify the identity of the proteins. Gel filtration standards (Bio-Rad) were run to calculate MW estimates.

### 4.4. Bacterial two-hybrid assays

To determine (self-) interactions, pKEK1286- and pKEK1287-derived constructs were subsequently transformed in to the *E. coli* reporter strain KDZif1ΔZ [70,101] and assayed for β-galactosidase activity. Experiments were performed in triplicate.

### 4.5. Helicase assays

Helicase activity was assayed by monitoring (and quantifying) the displacement of the radiolabelled (γ^32^P-ATP) 105-mer oligonucleotide oVP-1 (partially) annealed to the single-stranded circular DNA m13mp18 (ssM13; Affymetrix) essentially as previously described [57]. All reactions, containing 0.658 nM radiolabelled DNA substrate, were initiated by the addition of 2.5 mM ATP and carried out at 37°C in buffer containing 20 mM HEPES-NaOH (pH 7.5), 50 mM NaCl, 10 mM MgCl_2_ and 1 mM DTT for various lengths of time. In these reactions, proteins are in large excess (more than 250×) and thus not rate limiting. The reactions were terminated by adding 5× SDS-STOP buffer (100 mM Tris pH 8.0, 200 mM EDTA, 2.5% w/v SDS, 50% v/v glycerol, 0.15% (w/v) bromophenol blue). Proteins were added sequentially, with a 5 min preincubation after each addition. The gel was dried, scanned and analysed using a molecular imager and associated software (Bio-Rad). Experiments were carried out in triplicate, and data analysis was performed using Prism 6 (GraphPad Software).

### 4.6. RNA primer synthesis assay and thermally denaturing HPLC analysis

RNA priming assays and denaturing HPLC analyses were conducted as was previously described for other mesophilic bacterial primases [76]. The purified oligonucleotide templates used in this study were synthesized by Integrated DNA Technologies (Coralville, IA, USA). All RNA primer synthesis reactions were carried out in 50 µl nuclease-free water containing 50 mM HEPES pH 7.5, 100 mM potassium glutamate, 10 mM DTT, 2 µM ssDNA template, 30 mM magnesium acetate and 1.2 mM of each NTP, unless otherwise specified. For the denaturing HPLC analyses, a gradient 0–8.8% v/v acetonitrile over 16 min was used to obtain optimal separation of primer products and ssDNA templates on a WAVE HPLC nucleic acid fragment analysis system equipped with a DNA Sep HPLC column (Transgenomic; Omaha, NE, USA). The moles of RNA primers synthesized were quantified as previously described [56].

## Supplementary Material

Primase is required for helicase activity and helicase alters the specificity of primase in the enteropathogen Clostridium difficile - Smits et al. Supporting information
