# Supplementary material for: Primase is required for helicase activity and helicase alters the specificity of primase in the enteropathogen Clostridium difficile
Source: Open Biol. 2016 Dec 21;6(12):160272. doi: 10.1098/rsob.160272 (PMC5204125; doi:10.1098/rsob.160272)
Supplement: Primase is required for helicase activity and helicase alters the specificity of primase in the enteropathogen Clostridium difficile - Smits et al. Supporting information [file rsob160272supp1.pdf]

Supporting Information

Primase is required for helicase activity and helicase alters the specificity of primase in the enteropathogen *Clostridium difficile*

Erika van Eijk [1,#], Vasileios Paschalis [2,#], Matthew Green [2,#], Annemieke H. Friggen [1], Marilynn A. Larson [3,4], Keith Spriggs [5], Geoffrey S. Briggs [2], Panos Soultanas [2], Wiep Klaas Smits[1,*]

[1] Department of Medical Microbiology, Leiden University Medical Center, Leiden, the Netherlands; [2]School of Chemistry, Center for Biomolecular Sciences, University of Nottingham, United Kingdom; [3] Department of Pathology and Microbiology, University of Nebraska Medical Center, Omaha, NE 68198-5900, [4] National Strategic Research Institute, Omaha, NE 68105, [5] School of Psychology, University of Nottingham, United Kingdom.

Supporting Information table of contents

I. Supporting Materials and Methods

II. Supporting Figures

III. Supporting Tables

IV. Supporting References

I. Supporting Materials and Methods

Plasmid construction

All oligonucleotides and plasmids constructed for this study are listed in **Tables S1 and S2**.

To construct the CD3654, CD3657 and CD1454 expression plasmids, the open reading frames were amplified with high fidelity polymerase Pfu via PCR from *C. difficile* strain 630Δ*erm* chromosomal DNA [1,2], using primers oEVE-4 and oEVE-6 for CD3654, oWKS-1185 and oWKS-1367 for CD3657 and oWKS-1183 and oWKS-1184 for CD1454. The reverse primer in each PCR introduces a stop codon before the *Xho*I site, thereby ensuring that the protein is in its native form, when expressed (no C-terminal 6x His-tag). The CD3654 PCR product was digested with *Nco*I and *Xho*I restriction nucleases and ligated into vector pET28b (Novagen) to yield pEVE24. The CD3657 and CD1454 PCR products were digested with *Nde*I and *Xho*I and ligated into vector pET21b (Novagen) to yield pEVE87 and pEVE7, respectively. The DNA sequence of the constructs was verified by sequencing.

Construction of the plasmids for the bacterial two hybrid system was performed with Gateway cloning technology (Invitrogen) , which is based on phage λ site-specific recombination. To construct the CD3654 and CD3657 entry plasmids, the CD3654 and CD3657 open reading frames were amplified with high fidelity polymerase Pfu via PCR from *C.difficile* strain 630Δ*erm* chromosomal DNA, using primers oAF-26 and oAF-27 for CD3654 and oAF-28 and oAF-29 for CD3657. This resulted in *attB*-flanked PCR products (1 µl) that could be recombined into donor vector pDonR™201 (1 µl, 50 ng/ µl) with BP Clonase II enzyme mix (0.5 µl). The reaction was incubated at 25°C for 1.5 h and transformed into chemically competent *E. coli* DH5α cells by heat shock. After overnight incubation on LB plates at 37˚C, kanamycin

resistant colonies were selected. Bacterial two hybrid constructs were made by sub-cloning the genes of interest from the entry plasmids into the destination plasmids pKEK1286 (Zif fusion plasmid) or pKEK1287 (ω fusion plasmid) in an LR reaction [3]. In brief, the entry clones (1 µl, 50 ng/ µl) were mixed with one of the pKEK1286 or pKEK1287 (1 µl, 50 ng/ µl) destination vectors and LR Clonase II enzyme mix (0.5 µl). After the reaction was incubated at 25°C for 1.5 h, the mixture was transformed as a whole into chemically competent *E. coli* DH5α cells by heat shock. Resulting clones were selected with tetracycline for the Zif fusion plasmid or ampicillin for the ω fusion plasmid. The DNA sequence of all constructs (pEVE118, pEVE120, pEVE122, pEVE123, pEVE124 and pEVE125) was verified by sequencing.

Site-directed mutagenesis

Mutations in plasmids carrying CD3654, CD3654 and CD1454 mutants were constructed according to the QuikChange protocol (Stratagene). Primers were generated with Primer X, a web-based tool for automatic design of mutagenic primers for site-directed mutagenesis. QuikChange was carried out using Pfu polymerase and plasmids pEVE7, pEVE24, pEVE87, pEVE122, pEVE123, pEVE124 and pEVE125 as templates. Oligonucleotides used in the QuikChange reactions are listed in **Appendix Table S1**. All mutant constructs (**Appendix Table S2**) were verified for the correct mutation by DNA-sequencing.

Purification of the helicase CD3657

Overexpression *C. difficile* CD3657 was carried out in *E. coli* BL21 (DE3) from the pEVE-12 plasmid. The growth medium, consisting of 2xYT broth (1.2 L), carbenicillin (50 µg/mL) and

antifoam 204 (Sigma-Aldrich), was inoculated with a pre-culture (10 mL). The cell culture was incubated at 37°C with mechanical shaking at 180 rpm, until an optical density (600 nm) of 0.70-0.85 was reached (after approximately 3 h). Protein expression was induced via the addition of isopropyl β-D-1-thiogalactopyranoside (IPTG; 1 mM) and the culture was incubated at 30 °C for 3 h. The cells were harvested by centrifugation (3000 g, 15 min, 4 °C) and the resulting cell paste was stored at -80°C.

CD3657 cell paste, prepared from 1.2 L cell culture, was re-suspended in 30 mL TED50 buffer (Tris pH 7.5 50 mM, EDTA 1 mM, DTT 1 mM, NaCl 50 mM) with PMSF (1 mM). The bacterial cells were lysed by sonication and crude lysate was clarified by centrifugation (35,000 g, 30 min, 4°C). The resulting supernatant was separated from the cell debris using a 0.22 µm pore filter before a 50% w/v ammonium sulphate precipitation, followed by clarification by centrifugation (35,000 g, 30 min, 4°C). The ammonium sulphate precipitated pellet was suspended in TED50 buffer and loaded onto a 5 mL Q sepharose column, equilibrated in TED50 buffer. The protein was eluted using a gradient of 20 to 100% TED1000 buffer (Tris pH 7.5 50 mM, EDTA 1 mM, DTT 1 mM, NaCl 1000 mM) over 15 column volumes (CV). The fractions containing the protein of interest were pooled (12 mL, 25 mS) and diluted with 33 mL of TED50 buffer (Tris pH 7.5 50 mM, EDTA 1 mM, DTT 1 mM) to give an adjusted volume of 45 mL and conductivity of 10.1 mS. This solution was loaded onto a 5 mL heparin sepharose column equilibrated in TED50 buffer; the protein eluted in the flow through. After a further ammonium sulphate precipitation to concentrate the sample, the collected protein was loaded onto a Hiload 26/60 Superdex 200 gel filtration column equilibrated in TED50 buffer, yielding hexameric CD3657 oligomer. A further ammonium sulphate precipitation was used to

concentrate the sample in a reduced volume of 4 mL in TED50 buffer. Guanidinium chloride solution (8 M) was added in incremental steps (30 x 44.4 µL) to the protein solution with stirring, to give a final concentration of 2 M guanidinium chloride; this disrupts the oligomeric form of CD3657 but does not lead to complete protein unfolding. The protein was then loaded onto a Hiload 26/60 Superdex 200 gel filtration column equilibrated in TED50-GC buffer (Tris pH 7.5 50 mM, EDTA 1 mM, DTT 1 mM, NaCl 50 mM, guanidinium chloride 2 M), to give the CD3657 monomer. The protein was collected and the buffer was exchanged by dialysis against 2 L storage buffer (Tris pH 7.5 50 mM, EDTA 1 mM, DTT 1 mM, NaCl 50 mM, 20% v/v glycerol) for 18 h at 4°C.

The protein was quantified by UV spectrophotometry and stored at -80°C. Protein purity (>95%) was estimated by SDS-PAGE electrophoresis and concentration was determined spectrophotometrically using extinction coefficients calculated using the ExPASy ProtParam tool (http://web.expasy.org/protparam).

Walker A mutant proteins (K214R and T215A) were purified in a manner identical to the wild-type proteins after overexpression of the protein from the plasmids pEVE90 and pEVE92, respectively.

Purification of the putative loader protein CD3654

C. difficile CD3654 was expressed from the pEVE-24 plasmid in *E. coli* BL21 (DE3). The growth medium, consisting of 2xYT broth (1 L), kanamycin (30 µg/mL) and antifoam 204 (Sigma-Aldrich), was inoculated with a pre-culture (10 mL). The cell culture was incubated at 37 °C with mechanical shaking, until an optical density (600 nm) of 0.62-0.65 was reached (after

approximately 3 h). Protein expression was induced via the addition of IPTG (1 mM) and the culture was incubated at 30 °C for 3 h. The cells were harvested by centrifugation (3000 g, 15 min, 4 °C and the resulting cell paste was stored at -80 °C.

The bacterial cell paste, prepared from 1 L cell culture, was suspended in 25 mL TED50 buffer with PMSF (1 mM) and protease inhibitor cocktail (100 µL). The cells were lysed by sonication, clarified by centrifugation (40,000 g, 30 min, 4 °C) and the resulting supernatant separated from the cell debris using a 0.22 µm pore filter. Ammonium sulphate (7.32 g) was added slowly to the supernatant (25 mL) with stirring at 4 °C, to achieve 49% saturation. The protein pellet was collected by centrifugation (40,000 g, 30 min, 4 °C) and washed with TED20 buffer (Tris pH 7.5 50 mM, EDTA 1 mM, DTT 1 mM, NaCl 20 mM) (2 x 4 mL). The precipitate was suspended in TED20 buffer (15 mL) with gentle mechanical shaking (30 min, 4 °C). The suspension buffer was exchanged by dialysis against 1 L TED20 buffer for 2 h at 4 °C, giving a solution with conductivity of 9.5 mS. The protein solution (ca. 15 mL) was loaded onto combined 5 mL Q sepharose and 5 mL SP sepharose columns connected in series and equilibrated in TED20 buffer; the protein of interest eluted in the flow through. The collected protein was loaded onto a 5 mL heparin sepharose column equilibrated in TED20 buffer, and stepwise eluted to 15% TED1000 buffer. The collected protein was loaded onto a Hiload 26/60 Superdex 200 gel filtration column equilibrated in storage buffer (Tris pH 7.5 50 mM, EDTA 1 mM, DTT 1 mM, NaCl 50 mM, 10% v/v glycerol).

The protein was quantified by UV spectrophotometry and stored at -80 °C. Protein purity (>95%) was estimated by SDS-PAGE electrophoresis and concentration was determined

spectrophotometrically using extinction coefficients calculated using the ExPASy ProtParam tool (http://web.expasy.org/protparam).

Walker A (K198R, T199A) and B (D258Q) mutant proteins were purified in a manner identical to the wild-type proteins after overexpression of the protein from the plasmids pEVE59, pEVE60 and pEVE203, respectively.

Purification of the primase CD1454

Overexpression of *C. difficile* CD1454 was carried out in *E. coli* BL21 (DE3) from the pEVE7 plasmid. The growth medium consisting of 2xYT broth (1.2 L), carbenicillin (50 µg/mL) and antifoam 204 (Sigma-Aldrich) was inoculated with a pre-culture (10 mL). The cell culture was incubated at 37°C with mechanical shaking, at 180 rpm, until an optical density (600 nm) of 0.70-0.85 was reached (after approximately 3 h). Protein expression was induced via the addition of IPTG (1 mM final concentration) and the culture was incubated at 30°C for 3 h. The cells were harvested by centrifugation (3000 g, 15 min, 4 °C) and the resulting cell paste was stored at -80°C.

CD1454 cell paste, prepared from 1.2 L cell culture, was re-suspended in 30 mL TED0 sonication buffer (Tris pH 7.5 50 mM, EDTA 1 mM, DTT 1 mM) with PMSF (1 mM). The bacterial cells were lysed by sonication and crude lysate was clarified by centrifugation (42,000 g, 30 min, 4 °C). The resulting supernatant was separated from the cell debris using a 0.22 µm pore filter and loaded onto a 2x 5 ml HiTrap Q HP column in series with a 5 ml heparin sepharose column equilibrated in TED0 (50 mM Tris pH 7.5, 1 mM EDTA, 1 mM DTT). After loading, the columns were separated, and the heparin column was reconnected to the FPLC system, washed

extensively with TED0, step-washed with 10% TED1000 and gradient eluted with TED1000 (50 mM Tris pH 7.5, 1 mM EDTA, 1 mM DTT, 1000 mM NaCl). Primase eluted at 30-45 mS. Fractions containing primase were pooled, diluted with TED0 to 5-10 mS and loaded onto an 8 mL MonoS column equilibrated in TED0. The protein was eluted with a gradient of TED1000 and eluted at 15-20 mS. Fractions containing primase were pooled. The collected protein was loaded onto a Hiload 26/60 Superdex 200 gel filtration column equilibrated in TED100G buffer (50 mM Tris pH7.5, 1 mM EDTA, 1 mM DTT, 100 mM NaCl, 10% v/v glycerol). Protein aliquots were stored at -80 °C.

Protein purity (>95%) was estimated by SDS-PAGE electrophoresis and concentration was determined spectrophotometrically, using extinction coefficients calculated using the ExPASy ProtParam tool (http://web.expasy.org/protparam).

The lysine mutant CD1454 (K70H) was purified in a manner identical to the wild-type proteins after overexpression of the protein from plasmid pEVE201.

Gel-filtration experiments

Self-interaction of the CD3657 and CD3654 proteins were studied in the presence and absence of ATP. In brief, purified CD3657 (or mutant) or CD3654 (or mutant) was incubated for 10 min at room temperature with $MgCl_2$ (2 mM) in their storage buffer and ATP (1 mM). The mixture (500 µL) was loaded onto a Hiload 10/300 GL Superdex 200 analytical grade size exclusion column equilibrated in buffer A (Tris pH 7.5 50 mM, EDTA 1 mM, DTT 1 mM, glycerol 10% v/v, MgCl2 2 mM, ATP 1 mM) or buffer B (Tris pH 7.5 50 mM, EDTA 1 mM, DTT 1 mM, glycerol 10% v/v, MgCl2 2 mM) at a flow rate of 0.5 mL/min. The elution profiles from each experiment were

monitored at 280 nm and plotted as a function of the elution volume. Standards (Gel Filtration Standards 151-1901, BioRad) were run to allow MW estimation. The column resolved the largest protein (670kDa) from this standard as a peak at an elution volume of ~9mL.

To assess interactions between CD3657 and CD3654, purified proteins were mixed in a 1:1 stoichiometry in the presence of $MgCl_2$ (2 mM) and ATP (1 mM), and incubated for 10 min at room temperature. The mixture (500 µL) was loaded onto a Hiload 10/300 GL Superdex 200 analytical grade size exclusion column equilibrated in buffer A at a flow rate of 0.5 mL/min. For the experiments without ATP, buffer B was used.

Samples from fractions were analysed by SDS-PAGE and Coomassie Blue staining to verify the identity of the proteins.

Bacterial two-hybrid assays

To determine (self-) interaction, pKEK1286- and pKEK1287-derived constructs were subsequently transformed in to the *E. coli* reporter strain KDZif1ΔZ [3,4]. In order to control for background due to (possible) differences in expression of the constructs, single expression plasmids (Zif or ω fusion) containing the gene of interest were transformed into the reporter strain. After overnight incubation at 30˚C, three colonies per assay were cultured overnight in LB broth (30°C) in the presence of 1mM IPTG, tetracycline (selects Zif fusion plasmid) and/or ampicillin (selects ω fusion plasmid). Bacterial cells were permeabilized with SDS and chloroform and assayed for β- galactosidase activity. In short, cells were diluted in Z buffer (60 mM Na_2HPO_4, 40 mM NaH_2PO_4, 10 mM KCl, 1 mM $MgSO_4$, 50 mM β-mercaptoethanol, pH7) to 1 ml and permeabilized with 50 µl 0.1% w/v SDS and 100 µl chloroform. After 5 minutes of

equilibration, 200 µl of o-nitrophenyl-β-D-galactopyranoside was added to each tube and incubated at room temperature until yellow colour developed. The reaction was stopped with 0.5 ml 1 M Na_2CO_3 and measured at OD_{420nm} and OD_{550nm} to calculate the β- galactosidase activity in Miller Units. Experiments were performed in triplicate.

Helicase assays

Helicase activity was assayed by monitoring (and quantifying) the displacement of the radiolabelled (γ^{32}P-ATP) oligonucleotide oVP-1 (partially) annealed to the single stranded circular DNA m13mp18 (ssM13; Affymetrix) essentially as previously described [5]. In short, the 105-mer oligonucleotide was radiolabelled at the 5' end using γ^{32}P-ATP and T4 polynucleotide kinase (New England Biolabs) and subsequently purified through an S-200 mini-spin column (GE Healthcare). All reactions, containing 0.658 nM radiolabelled DNA substrate, were initiated by the addition of 2.5 mM ATP and carried out at 37˚C in buffer containing 20 mM HEPES-NaOH (pH 7.5), 50 mM NaCl, , 10 mM $MgCl_2$ and 1 mM DTT for various times. The reactions were terminated by adding 5x SDS-STOP buffer (100 mM Tris pH8.0, 200 mM EDTA, 2.5% w/v SDS, 50% v/v glycerol, 0.15% (w/v) bromophenol blue).

To investigate the effect of the putative helicase loader CD3654 or primase CD1454 on the activity of the helicase CD3657, the proteins were mixed in equimolar concentrations (1 µM) and incubated for 5 minutes at 37˚C before initiating the reaction with 2.5 mM ATP (final concentration). For three protein reactions, the buffer with CD3657 was preincubated for 5 mins before the addition of CD3654, then further preincubated for 5 mins 37˚C , followed by

the addition of CD1454 and further preincubation, prior to initiation of the reaction with 2.5 mM ATP.

Stop buffer was added to terminate the reactions (1% v/w SDS, 40 mM EDTA, 8% v/v glycerol, 0.1% w/v bromophenol blue). Reaction samples (10 µl) were loaded on a 10% non-denaturing polyacrylamide gel, run in 1xTBE (89 mM Tris, 89 mM boric acid, 2 mM EDTA) at 150V, 40mA/gel for 60 mins. The gel was dried, scanned and analyzed using a molecular imager and associated software (Biorad). Experiments were carried out in triplicate, and data analysis was performed using Prism 6 (GraphPad Software).

Size exclusion chromatography coupled with multiangle laser light scattering analysis

The oligomeric state of CD1454 protein was assessed using an 1260 Infinity HPLC system (Agilent), with a miniDAWN Treos 3-angle static light scattering detector (Wyatt Technologies) and ERC RefractoMax 521 UV_{280nm} and refractive index detector (ThermoScientific), connected downstream of a Superose 6 10/300 gel filtration column (GE Healthcare) equilibrated in 50 mM Tris, pH 7.5, 50 mM NaCl, 1 mM EDTA, 1 mM DTT, 1 mM ATP, 2 mM $MgCl_2$. The data was processed using the Astra™ package version 6.1 (Wyatt Technologies).

RNA primer synthesis assay and thermally denaturing HPLC analysis

RNA priming assays and denaturing HPLC analyses were conducted as was previously described for other mesophilic bacterial primases [6]. Initially, two 50-mer oligonucleotides that comprised all 64 trinucleotide sequences were used to assess enzymatic activity and template specificity, as previously described [7]. Confirmation of the preferred initiation motif was

obtained by using the 23-mer trinucleotide-specific template 5'-CAGA(CA)5XYZ(CA)3-1,3-propanediol, whereby XYZ was the trinucleotide of interest. The purified oligonucleotide templates used in this study were synthesized by Integrated DNA Technologies, Inc. (Coralville, IA). Templates were quantified by spectrophotometry at 260 nm using the extinction coefficients obtained for each oligonucleotide from the online OligoTools Calculator from Integrated DNA Technologies. All RNA primer synthesis reactions were carried out in 50 µL nuclease-free water containing 50 mM HEPES pH 7.5, 100 mM potassium glutamate, 10 mM DTT, 2 µM ssDNA template, 30 mM magnesium acetate, and 1.2 mM of each NTP, unless otherwise specified. Priming reactions were incubated at 30°C for 1 h, desalted on a Sephadex G-25 spin column, and dried using a speed vacuum. The pellet was suspended in water to 1/5th of the original volume of the sample and 8 µL of that sample was analyzed by HPLC under thermally-denaturing conditions at 80°C.

For the denaturing HPLC analyses, a gradient 0–8.8% v/v acetonitrile over 16 min was used to obtain optimal separation of primer products and ssDNA templates on a DNA Sep column. The WAVE HPLC Nucleic Acid Fragment Analysis System, HPLC Buffer A (0.1 M triethylammonium acetate, pH 7.0), HPLC Buffer B (0.1 M triethylammonium acetate, 25% v/v acetonitrile), and the DNA Sep HPLC column were obtained from Transgenomic (Omaha, NE). Primer products and ssDNA templates were detected by UV absorbance at 260 nm and elution times of the nucleic acids were correlated to the elution profile of the appropriate standard to confirm composition and length. To normalize variability introduced during sample preparation and injection into the HPLC column, primer abundance was determined by using the ssDNA template as an internal standard. The moles of RNA primers synthesized were quantified using

the relative extinction coefficient for the oligonucleotides and reported as the sum of moles for all primer lengths, as previously described [8].

II. Supporting Figures

Figure S1. Phyre2 model of the CD1454 primase. The model is based on PDB 2AU3. Domains are colored as follows. Zinc finger (blue; zf-CHC2/PFAM01807), catalytic domain (green: TOPRIM_N/PFAM8275; yellow: TOPRIM_DnaG_primases/cd03364) and helicase interacting domain (red: DnaB_bind; PFAM10410).

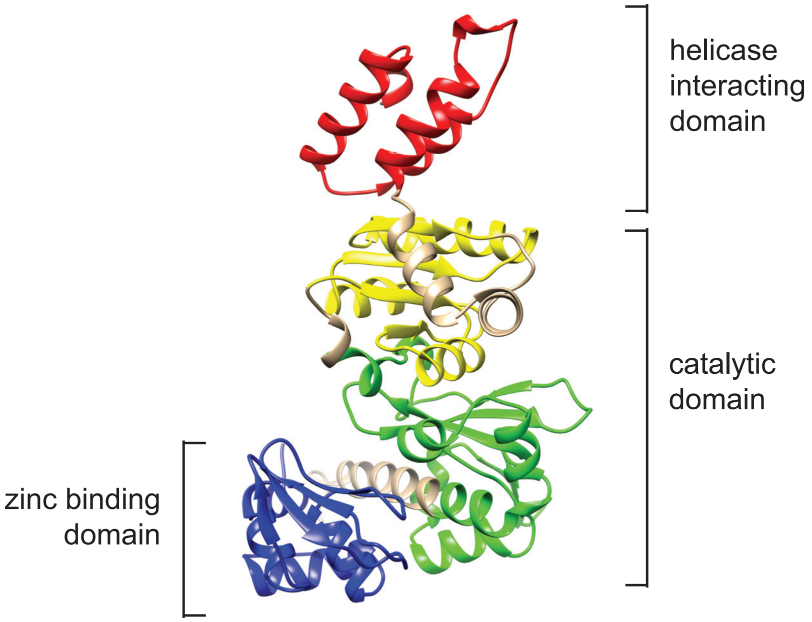

Figure S2. CD3657 demonstrates concentration dependent hexamerisation. Analytical gel filtration was performed in buffer B (Tris pH 7.5 50 mM, EDTA 1 mM, DTT 1 mM, glycerol 10% v/v, MgCl2 2 mM) on a HiLoad 10/300 GL Superdex 200 analytical grade size exclusion column with the indicated concentration of CD3657 protein.

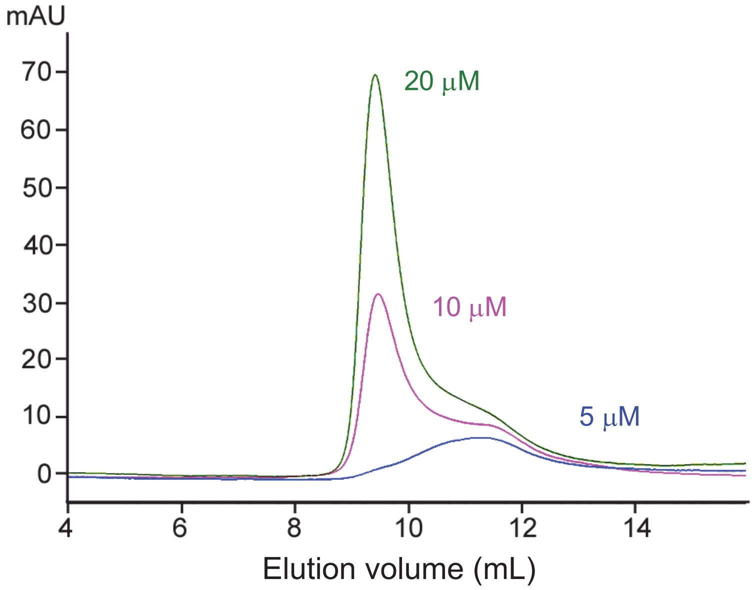

Figure S3. ATP dependent interaction of the helicase CD3657 and the putative loader CD3654 at high concentrations of proteins. The helicase CD3657 and the putative loader CD3654 interact in an ATP-dependent manner. Analytical gel filtration was performed in the presence (red) of absence (green) of 1mM ATP on a Hiload 10/300 GL Superdex 200 analytical grade size exclusion column. Inset shows Coomassie stained SDS-PAGE gels of sampled fractions.

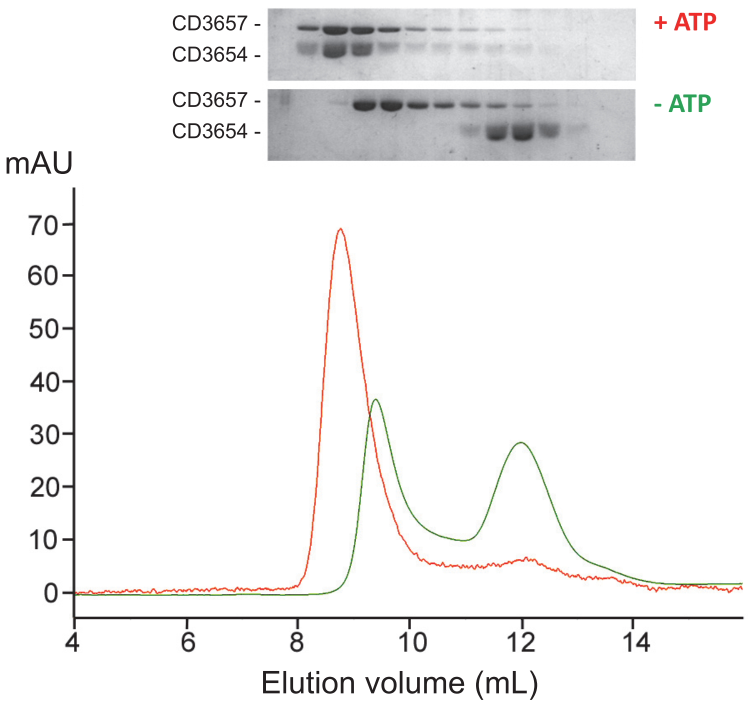

Figure S4. Walker A mutants of CD3654 retain the ability to interact with CD3657. **A**. Bacterial two hybrid analysis of the interaction between CD3657 and CD3654 K198R. **B**. Bacterial two hybrid analysis of the interaction between CD3657 and CD3654 T199A. Bar graphs in A-B indicate average values and error-bars indicate standard deviation of the measurements (n=3). Significance was determined using the Student's t-test (* p< 0.05, ** p<0.001). **C**. Analytical gel filtration analysis of the interaction between CD3657 and CD3654 K198R. **D**. Analytical gel filtration analysis of the interaction between CD3657 and CD3654 T199A. Analytical gel filtration was performed in buffer B (see Methods) with 3.10 µM of each protein in the presence of 1 mM ATP. Inset in C-D shows a Coomassie stained SDS-PAGE gel of the numbered peak fractions.

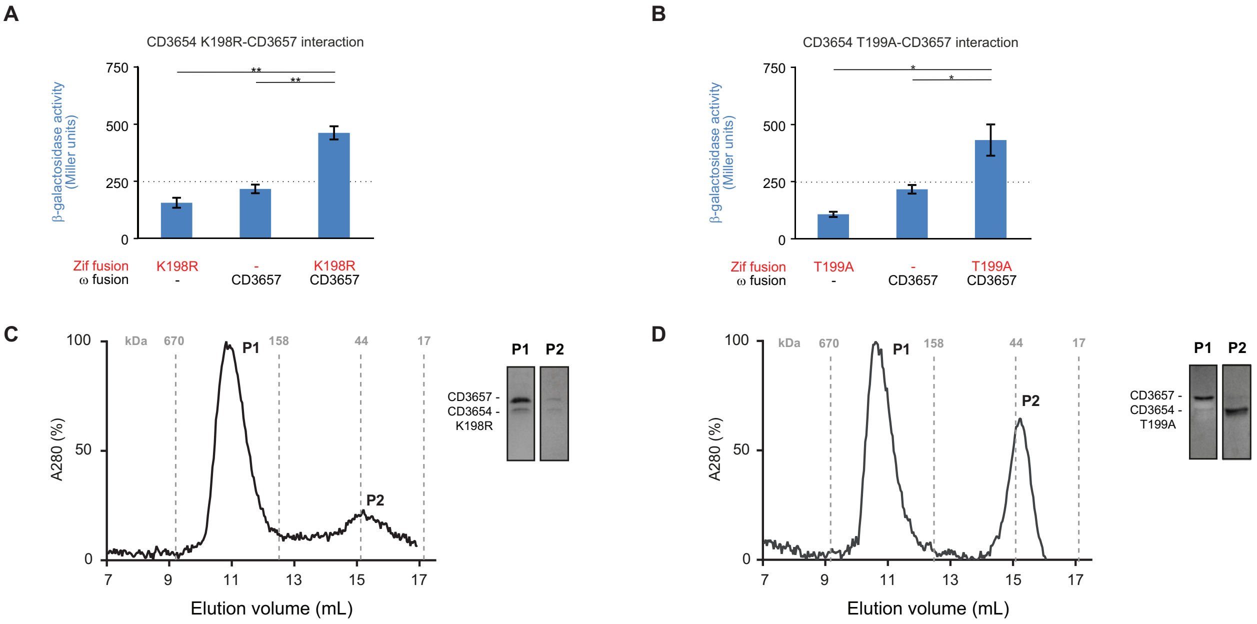

Figure S5. Walker A mutants of CD3654 are monomeric.

A. Bacterial two hybrid analysis of the self-interaction of CD3654 K198R. **B**. Bacterial two hybrid analysis of the self-interaction of CD3654 T199A. Bar graphs in A-B indicate average values and error-bars indicate standard deviation of the measurements (n=3). Significance was determined using the Student's t-test (* $p < 0.05$, ** $p < 0.001$).

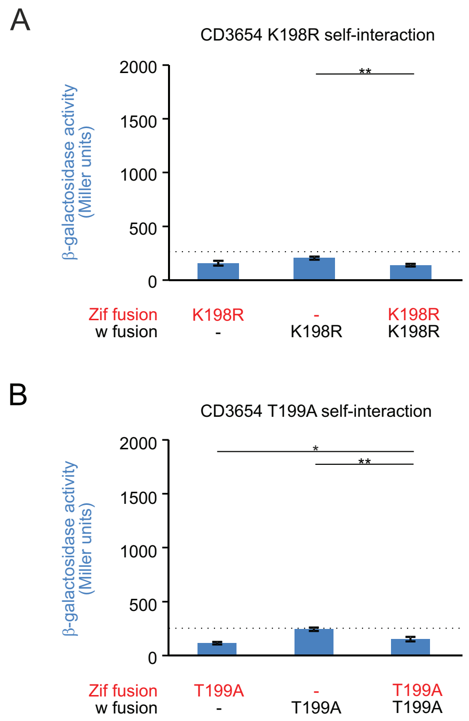

Figure S6. Helicase activity in the presence of helicase and (putative) loader proteins of *B. subtilis* and *C. difficile*. Helicase activity was assayed by quantifying the displacement of a radiolabelled (γ^{32}P-ATP) oligonucleotide partially annealed to the single stranded circular DNA m13mp18. Percent displaced signal from the helicase assays in time for the *B. subtilis* proteins or *C. difficile* proteins.

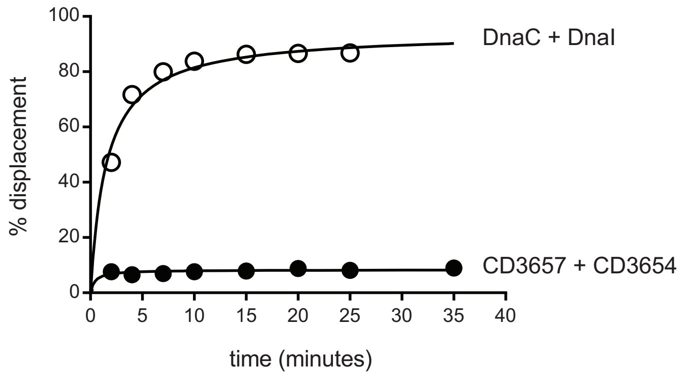

Figure S7. Molecular mass determination of the CD1454 primase protein. Indicated molecular mass from SEC-MALS experiments is calculated based on light scattering (LS) and dRI signals at the peak of the curve (Mp 72.9 kDa/ 0.5% uncertainty, Mw 74.1 kDa/0.9% uncertainty). The molecular mass indicates a monomeric form of the protein.

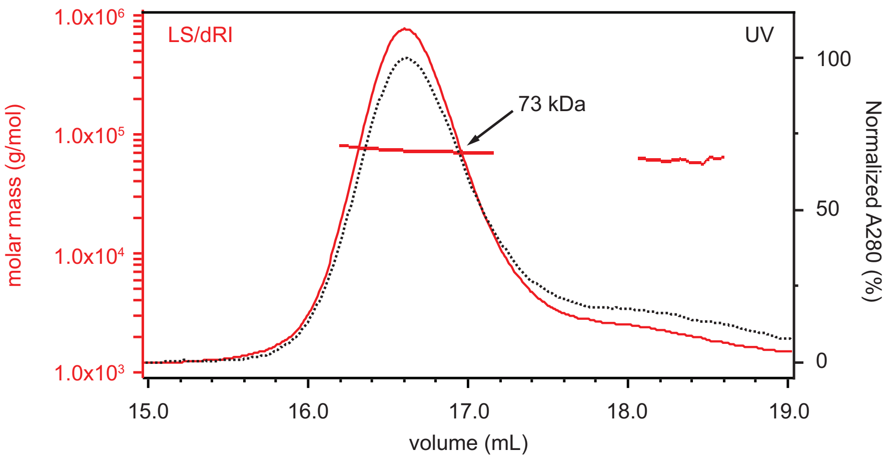

Figure S8. Helicase activity is abrogated in a Walker A mutant of CD3657. Helicase activity was assayed by quantifying the displacement of a radiolabelled (γ^{32}P-ATP) oligonucleotide partially annealed to the single stranded circular DNA m13mp18. Percent displaced signal from the helicase assays in time. Helicase activity of the wild type (WT; closed black circles) and mutant (K214R and T215A; open and closed red circles, respectively) CD3657 proteins in the presence of the CD1454 primase. Error bars indicate standard deviation (N=3).

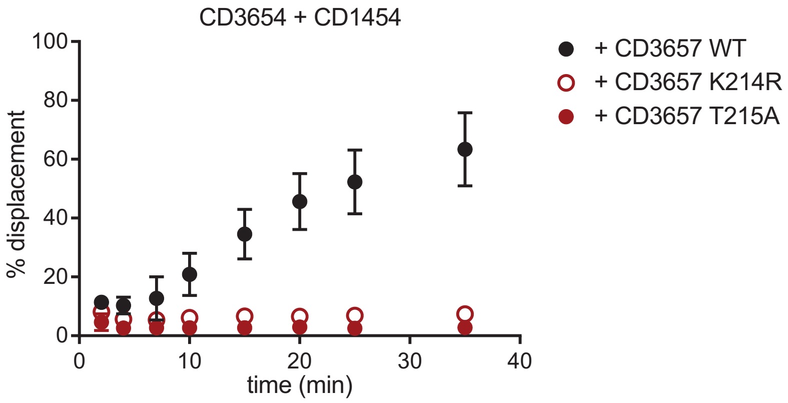

Figure S9. Helicase activity is not inhibited in the presence of mutant CD3654 loader proteins.

A. Helicase activity of CD3657 in the presence of a Walker A mutant CD3654 protein (K198R), with (black) and without (red) the CD1454 primase protein. **B**. Helicase activity of CD3657 in the presence of Walker B mutant CD3654 protein (D258Q), with (black) and without (red) the CD1454 primase protein. Error bars indicate standard deviation (N=3).

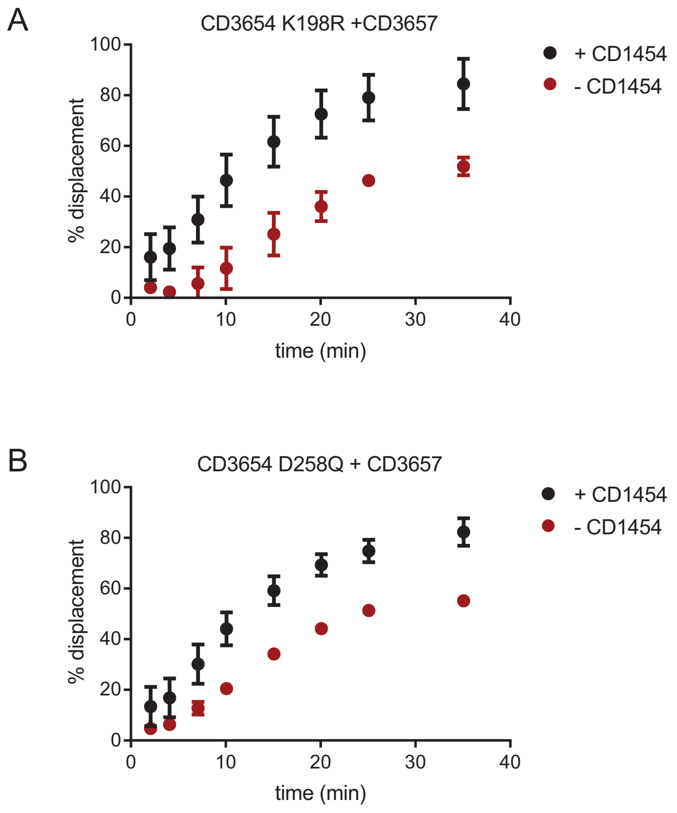

Figure S10. Effect of Mg2+ and NTP concentration on CD1454 activity. Primase activity was determined using the RNA primer synthesis assay and thermally denaturing high-performance liquid chromatography analysis. **A**. Primase activity as a function of magnesium ion concentration. Shown are the relative levels of RNA primers synthesized CD1454 (1.8 µM) in reactions with the 5’-d(CCC)-containing ssDNA template and the indicated concentration of magnesium acetate. **B**. Primase activity as a function of NTP concentration. Shown are the relative levels of RNA primers synthesized by CD1454 (1.8 µM) in reactions with 20 mM magnesium acetate, the 23-mer 5’-d(CCC)-containing ssDNA template, and the indicated concentration of each ribonucleotide.

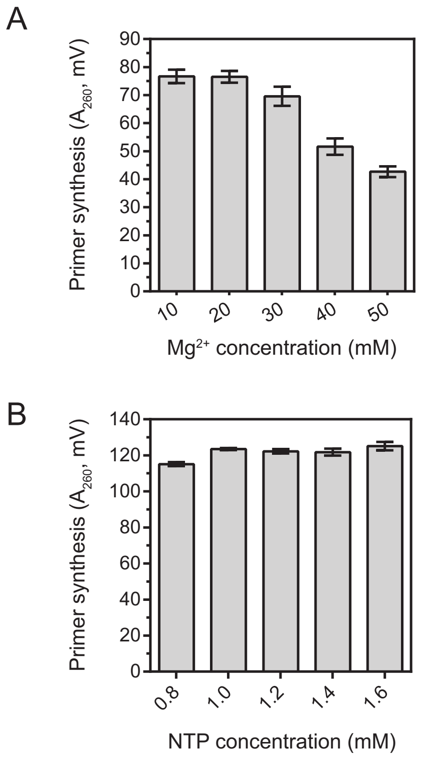

Figure S11. **The effect of the 5' flanking base on priming efficiency of CD1454 at the preferred trinucleotide**. Primase activity was determined by thermally denaturing high-performance liquid chromatography analysis on templates with the preferred 5' d(CCC) trinucleotide and varying 5' flanking bases. The numbers in the panel to the right of the chromatograms denote the total peak area for the RNA primer products synthesized and shown in the associated chromatogram to the left.

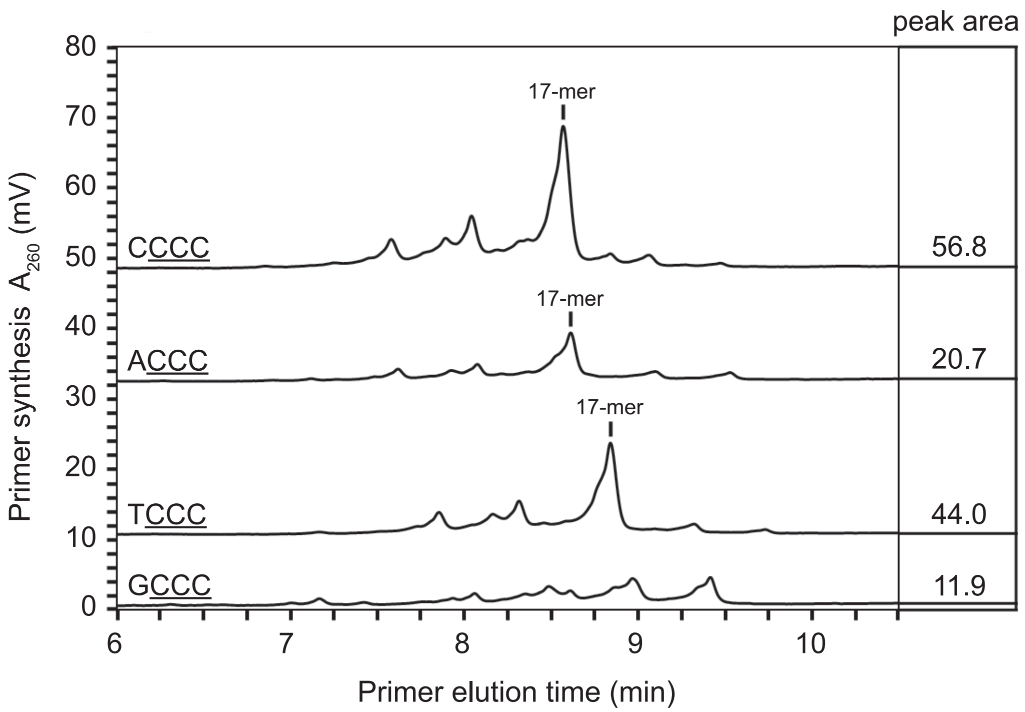

III. Supporting Tables.

Table S1. Oligonucleotides used in this study

Name	Sequence (5' – 3')	Description
oAF-26	GGGGACAAGTTTGTACAAAAAAGCAGGCTTAATGAATGAGGATAAAATAAGAAAAATAC	Forward CD3654 B2H
oAF-27	GGGGACCACTTTGTACAAGAAAGCTGGGTCTTTAAATCTTTCCCATCTCAAATCATC	Reverse CD3654 B2H
oAF-28	GGGGACAAGTTTGTACAAAAAAGCAGGCTTAATGGAAGATATGACGAGAATTCCTC	Forward CD3657 B2H
oAF-29	GGGGACCACTTTGTACAAGAAAGCTGGGTCTAGGTCTCTCAACTTATCCCC	Reverse CD3657 B2H
oEVE-4	GCTTCCATGGATGAGGATAAAATAAGAAAAATACTTGC	Forward CD3654 pET
oEVE-6	GACGCTCGAGTTATTTAAATCTTTCCCATCTCAAATCATCTCC	Reverse CD3654 pET
oWKS-1185	GTGATTCATATGGAAGATATGACGAG	Forward CD3657 pET
oWKS-1367	CCGCTCGAGTTATAGGTCTCTCAACTTATCCCC	Reverse CD3657 pET
oWKS-1183	TAGAATACATATGTTAACACAAAAAATTACACCAG	Forward CD1454 pET
oWKS-1184	CCGCTCGAGTTACTACAAGCTCTTTAAAATTTTATTTATC	Reverse CD1454 pET
oWKS-1272	GGTTCTACTGGACTAGGAAGGACCTATATGTGCAATTG	Forward CD3654 K198R QuikChange
oWKS-	CAATTGCACATATAGGTCCTTCCTAGTCCAGTAGAA	Reverse CD3654 K198R QuikChange

1273	CC	
oWKS-1274	GGTTCTACTGGACTAGGAAAGGCCTATATGTGCAATTGTATTG	Forward CD3654 T199A QuikChange
oWKS-1275	CAATACAATTGCACATATAGGCCTTTCCTAGTCCAGTAGAACC	Reverse CD3654 T199A QuikChange
oEVE-69	CTTGTTTGATTGTGATTTATTAATAATACAAGACCTTGGAAC	Forward CD3654 D258Q QuikChange
oEVE-70	GTTCCAAGGTCTTGTATTATTAATAAATCACAATCAAACAAG	Reverse CD3654 D258Q QuikChange
oWKS-1276	GCTAGACCAGCAATGGGTAGAACTGCCTTTTCTTTAAAC	Forward CD3657 K214R QuikChange
oWKS-1277	GTTTAAAGAAAAGGCAGTTCTACCCATTGCTGGTCTAGC	Reverse CD3657 K214R QuikChange
oWKS-1278	CTAGACCAGCAATGGGTAAAGCTGCCTTTTCTTTAAACTTGG	Forward CD3657 T215A QuickChange
oWKS-1279	CCAAGTTTAAAGAAAAGGCAGCTTTACCCATTGCTGGTCTAG	Reverse CD3657 T215A QuickChange
oEVE-77	CGTCAAAACAGATTTATCACTGTTTTGGTTGTGGTG	Forward CD1454 K70H QuikChange
oEVE-78	CACCACAACCAAAACAGTGATAAATCTGTTTTGACG	Reverse CD1454 K70H QuikChange
oVP-1	CACACACACACACACACACACACACACACACACACACACACACACACACACACACACCCCCTTTAAAAAAAAAAGCCAAAAGCAGTGCCAAGCTTGCATGCC	Helicase assay

Table S2. Plasmids used in this study.

Plasmid	Description	Reference
pEVE24	pET28b-CD3654	This study
pEVE59	pET28b-CD3654 K198R	This study
pEVE60	pET28b-CD3654 T199A	This study
pEVE-203	pET28b-CD3654-D258Q	This study
pEVE87	pET21b-CD3657	This study
pEVE90	pET21b-CD3657 K214R	This study
pEVE92	pET21b-CD3657 T215A	This study
pEVE-7	pET21b-CD1454	This study
pEVE-201	pET21b-CD1454-K70H	This study
pEVE118	pENTRY-CD3654	This study
pEVE120	pENTRY-CD3657	This study
pEVE122	pKEK1286-CD3654	This study
pEVE123	pKEK1287-CD3654	This study
pEVE124	pKEK1286-CD3657	This study
pEVE125	pKEK1287-CD3657	This study
pEVE167	pKEK1286-CD3654 K198R	This study
pEVE168	pKEK1286-CD3654 T199A	This study
pEVE169	pKEK1287-CD3654 K198R	This study
pEVE170	pKEK1287-CD3654 T199A	This study
pEVE171	pKEK1286-CD3657 K214R	This study
pEVE172	pKEK1286-CD3657 T215A	This study
pEVE173	pKEK1287-CD3657 K214R	This study
pEVE174	pKEK1287-CD3657 T215A	This study

Supporting References

1. Hussain HA, Roberts AP, Mullany P (2005) Generation of an erythromycin-sensitive derivative of Clostridium difficile strain 630 (630Deltaerm) and demonstration that the conjugative transposon Tn916DeltaE enters the genome of this strain at multiple sites. J Med Microbiol 54: 137-141.

2. van Eijk E, Anvar SY, Browne HP, Leung WY, Frank J, Schmitz AM, Roberts AP, Smits WK (2015) Complete genome sequence of the Clostridium difficile laboratory strain 630Deltaerm reveals differences from strain 630, including translocation of the mobile element CTn5. BMC Genomics 16: 31. 10.1186/s12864-015-1252-7 [doi];s12864-015-1252-7 [pii].

3. Karna SL, Zogaj X, Barker JR, Seshu J, Dove SL, Klose KE (2010) A bacterial two-hybrid system that utilizes Gateway cloning for rapid screening of protein-protein interactions. Biotechniques 49: 831-833. 000113539 [pii];10.2144/000113539 [doi].

4. Vallet-Gely I, Donovan KE, Fang R, Joung JK, Dove SL (2005) Repression of phase-variable cup gene expression by H-NS-like proteins in Pseudomonas aeruginosa. Proc Natl Acad Sci U S A 102: 11082-11087. 0502663102 [pii];10.1073/pnas.0502663102 [doi].

5. Rannou O, Le CE, Larson MA, Nouri H, Dalmais B, Laughton C, Janniere L, Soultanas P (2013) Functional interplay of DnaE polymerase, DnaG primase and DnaC helicase within a ternary complex, and primase to polymerase hand-off during lagging strand DNA replication in Bacillus subtilis. Nucleic Acids Res 41: 5303-5320. gkt207 [pii];10.1093/nar/gkt207 [doi].

6. Larson AM, Fung AM, Fang FC (2010) Evaluation of tcdB real-time PCR in a three-step diagnostic algorithm for detection of toxigenic Clostridium difficile. J Clin Microbiol 48: 124-130.

7. Larson MA, Bressani R, Sayood K, Corn JE, Berger JM, Griep MA, Hinrichs SH (2008) Hyperthermophilic Aquifex aeolicus initiates primer synthesis on a limited set of trinucleotides comprised of cytosines and guanines. Nucleic Acids Res 36: 5260-5269. gkn461 [pii];10.1093/nar/gkn461 [doi].

8. Koepsell SA, Larson MA, Frey CA, Hinrichs SH, Griep MA (2008) Staphylococcus aureus primase has higher initiation specificity, interacts with single-stranded DNA stronger, but is less stimulated by its helicase than Escherichia coli primase. Mol Microbiol 68: 1570-1582. MMI6255 [pii];10.1111/j.1365-2958.2008.06255.x [doi].
